# Identification of kukoamine a as an anti-osteoporosis drug target using network pharmacology and experiment verification

**DOI:** 10.1186/s10020-023-00625-6

**Published:** 2023-03-20

**Authors:** Liying Luo, Zhiyuan Guan, Xiao Jin, Zhiqiang Guan, Yanyun Jiang

**Affiliations:** 1 Department of Ophthalmology, Tongren Hospital, Shanghai Jiao Tong University School of Medicine, Shanghai, China; 2 Department of Orthopedics, The Shanghai Tenth People’s Hospital of Tongji University, Shanghai, China; 3 Department of Rheumatology and Immunology, The First People’s Hospital of Xuzhou, Xuzhou, Jiangsu 221002 People’s Republic of China; 4 Department of Dermatology, Xuzhou Municipal Hospital Affiliated With Xuzhou Medical University, Xuzhou, Jiangsu 221002 People’s Republic of China

**Keywords:** Osteoporosis, Kukoamine A, Osteoblast cells, Ovariectomized mice, Network pharmacology

## Abstract

**Background:**

Osteoporosis (OP) is a major and growing public health problem characterized by decreased bone mineral density and destroyed bone microarchitecture. Previous studies found that Lycium Chinense Mill (LC) has a potent role in inhibiting bone loss. Kukoamine A (KuA), a bioactive compound extract from LC was responsible for the anti-osteoporosis effect. This study aimed to investigate the anti-osteoporosis effect of KuA isolated from LC in treating OP and its potential molecular mechanism.

**Method:**

In this study, network pharmacology and molecular docking were investigated firstly to find the active ingredients of LC such as KuA, and the target genes of OP by the TCMSP platform. The LC-OP-potential Target gene network was constructed by the STRING database and network maps were built by Cytoscape software. And then, the anti-osteoporotic effect of KuA in OVX-induced osteoporosis mice and MC3T3-E1 cell lines were investigated and the potential molecular mechanism including inflammation level, cell apoptosis, and oxidative stress was analyzed by dual-energy X-ray absorptiometry (DXA), micro-CT, ELISA, RT-PCR, and Western Blotting.

**Result:**

A total of 22 active compounds were screened, and we found KuA was identified as the highest active ingredient. Glycogen Phosphorylase (*PYGM*) was the target gene associated with a maximum number of active ingredients of LC and regulated KuA. In vivo, KuA treatment significantly increased the bone mineral density and improve bone microarchitecture for example increased BV/TV, Tb.N and Tb.Th but reduced Tb.Sp in tibia and lumber 4. Furthermore, KuA increased mRNA expression of osteoblastic differentiation-related genes in OVX mice and protects against OVX-induced cell apoptosis, oxidative stress level and inflammation level. In vitro, KuA significantly improves osteogenic differentiation and mineralization in cells experiment. In addition, KuA also attenuated inflammation levels, cell apoptosis, and oxidative stress level.

**Conclusion:**

The results suggest that KuA could protect against the development of OP in osteoblast cells and ovariectomized OP model mice and these found to provide a better understanding of the pharmacological activities of KuA again bone loss.

**Supplementary Information:**

The online version contains supplementary material available at 10.1186/s10020-023-00625-6.

## Introduction

Osteoporosis (OP) is a major and growing public health problem characterized by decreased bone mineral density and destroyed bone microarchitecture (Silverstein et al. 2021). With the inevitable consequence of aging, osteoporotic fractures are becoming more and more common in women over 55 and men over 65, causing huge costs in mortality and health care (Compston et al. 2019). Ironically, despite great advances in the treatment of OP, treatment gaps varied widely for patients at high risk for OP fractures both between and within countries (Khosla and Hofbauer 2017). Considering the complicated mechanisms and different treatment effects of OP, a large and growing body of literature showed that anti-osteoporosis targets have become a promising research field in recent years.

In China, many traditional Chinese medicines are used to treat diabetes and have been proven to be effective. For example, Lycium chinense Mill. (Lycii Cortex, LC) has great potential in preventing diabetes and glucocorticoid‑induced bone loss (Lee et al. 2021; Park et al. 2019a). However, relatively little information is available on the properties of potential hypoglycemic compounds of LC (Liu et al. 2021). Kukoamine A (KuA), a sperm kaloid, is a critical bioactive component extracted from the root bark of LC. KuA has several pharmacological effects such as anti-inflammatory, anti-pain, antibacterial, neuroprotective, autoimmune enhancing, and hypotensive effects (Hadjipavlou-Litina et al. 2009). Other bioactive component such as betaine, scoplin, Kukoamine B extracted from LC also showed significant effectiveness in the treatment of OP (Lee et al. 2021; Park et al. 2019a, 2020; Yajun et al. 2021). However, a systematic investigation of the main bioactive components of LC that contributed to OP remains unexplored.

To date, network pharmacology integrates pharmacological, bioinformatics, and other scientific analyses into a systematic network and interprets the therapeutic mechanisms of different drug components and the targets of gene delivery. Network pharmacology is a promising approach for understanding multicomponent drug systems such as Traditional Chinese Medicine (TCM) formulae (Li and Zhang 2013). By analyzing the components and targets of diseases, we can provide biological processes and pathways that TCM may play a role in, helping us to analyze the mechanism of TCM treating diseases. Molecular docking is a drug development method that mimics the interaction between receptors and drugs. In recent years, the use of molecular docking to elucidate the appropriate mechanism has become a global trend in drug development (Zhou et al. 2021a). Molecular function and signaling pathways by constructing a “disease-phenotype-genetic’ network can suitably interpret the relationship among different bioactive components in traditional Chinese medicine (Wei et al. 2020; Zhang et al. 2019a).

The current study aimed to investigate the relationship between the potential bioactive components in LC with OP. In this regard, we firstly used network pharmacology to analyze the effective ingredients of LC, then screened the bioactive ingredients in the treatment of OP. These selected targets were evaluated by pathways of action in functional enrichment pharmacology, genetic selection (GO), biological pathways (KEGG), and molecular docking technology (Jiang et al. 2019). Finally, the ovariectomized OP mice with different doses of KuA and gene silencing experiments at the cell level further verified the results of network pharmacologic analysis. This study provided a theoretical basis for investigating the molecular mechanism of LC against OP. The workflow is shown in Additional file 1: Figure S1.

## Materials and methods

### Ovariectomized OP model mice with different doses of KuA

The experimental protocol was approved by the Department of Laboratory Animal Science of the Shanghai Tenth People’s Hospital of Tongji University (SHDSYY-2021–6420, data:2021.5.6). A total of forty-five C57BL/6N female mice (8 weeks) were housed in the same animal room with a controlled temperature (22 °C) and light cycle (12 h light, 12 h dark) with free access to fresh water and food. The mice were divided randomly into five groups (n = 9): (1) Sham (n = 9), (2) Ovariectomized (OVX, n = 9), (3) OVX + 5 mg/kg/day of KuA (KuA5, n = 9), (4) OVX + 10 mg/kg/day of KuA (KuA10, n = 9), (5) OVX + 20 mg/kg/day of KuA (KuA20, n = 9). The mice treated with osteoporotic intervention measures were administered one week after surgery (Zheng et al. 2020) during 11 weeks of administration. The bone mineral density (BMD) of the right tibia and spine was measured at 0, 6, and 12 weeks respectively after ovariectomy (0 weeks is the time of ovariectomy). All operations were performed to minimize animal suffering and reduce the number of mice used. KuA (purity ≧98%, Liaoning University, Shenyang, China) was dissolved in DMSO and administered by the intravenous route as in previous studies (Liu et al. 2017). All related reagents were of analytical or pharmaceutical grade.

Each mouse used in these studies was euthanized with pentobarbital (50 mg/kg, intraperitoneal injection). Meanwhile, the protocol of mice sham surgery or bilateral oophorectomy was described as before (Inada et al. 2011). All mice underwent intraperitoneal anesthesia with the injection of pentobarbital (50 mg/kg), and the sham group was exposed to both sides of the ovary and raised the fatty tissue around the ovary, leaving the ovary intact, but bilateral ovariectomy for the other eighteen mice performed under the premise of complete ovarian exposure resection. After the surgery, intraperitoneal injection of penicillin was used to prevent infection twice a day for two days.

### Dual-energy X-ray absorptiometry (DXA) and Micro-CT analysis

At the end of 12 weeks after treatment, Lumbar spine (L4) and right tibia bone mineral density (BMD) were determined in small animals using a high-resolution soft X-ray collimator (Faxitron® LX-60 Cabinet radiography system, US). The sample was then placed on the base of the PBS scanner using μ-CT (Inveon, Siemens, Erlangen, Germany) at a spatial resolution of 55 kVp, 145 uA, integration time 300 ms, 720 views, 20 mm voxel resolution. The region of interest (ROI) was 0.36 to 2.1 mm from the right proximal epiphyseal growth plate of the tibia, 1.5 mm long, and 0.5 mm from the L4 proximal growth plate. We analyzed trabecular and cortical bone microarchitecture by measuring bone volume (BV) over total volume (TV), trabecular thickness (Tb.Th), trabecular number (Tb.N), trabecular spacing (Tb.Sp) in the medial tibial trabecular bone and lumbar vertebrae, the total cross‐sectional area inside the periosteal envelope (Tt.Ar), cortical bone area (Ct.Ar), bone marrow area (Ma.Ar), average cortical thickness (Ct.Th) and cortical area fraction (Ct.Ar/Tt.Ar) in the medial tibial cortical bone which has been describing in previous studies (Kalyanaraman et al. 2017; Lei et al. 2018). μ-CT did not analyze surrounding osteophytes.

### Biomechanical analysis

Following radiographic measurements, three-point flexion testing was performed on these right tibias using a mechanical testing system (Landmark, MTS, Inc., Eden Prairie, MN) to determine the mechanical properties. The main support section was 9 mm and the load range was 5 mm. The tibia was placed in a bracket with a stretched medial surface, and the distal portion of the tibiofibular junction was placed directly into the leftmost fixation device. Each tibia should be loaded at 0.01 mm/s until rupture and the load and displacement recorded. Data was automatically recorded by the material testing device. According to the load–displacement curve, the biomechanical properties were evaluated to analyze compressive maximum load [a measure of the maximum force that the sample tibia withstood before fracture (N)], stiffness [the slope on the linear portion of the load-deformation curve related to the tibia's flexural rigidity (N/mm)], displacement of maximum force [a measure of the maximum displacement that the sample tibia withstood before fracture (mm)] and the energy of maximum force (area under the linear portion of the load-deformation curve (mJ) [the slope on the linear portion of the load-deformation curve related to the tibia's flexural rigidity (N/mm)].

### Western Blotting

To analyze the oxidative stress level, we extracted mitochondrial and cytosolic proteins. The mitochondria were isolated from bone tissue using a Mitochondria Isolation Kit (QuadroMACS 130–094-532) according to the manufacturer’s instructions. Other proteins were extracted from bone tissue, and quantitated with a protein assay kit (Bio-Rad, Mississauga, Ontario, Canada). Protein samples (15 µg) were fractionated by SDS-PAGE and transferred to nitrocellulose membranes. Protein concentration was quantified using the BCA Reagent (Thermo Scientific, XH351428).

We performed western blotting analysis with rabbit anti-caspase-3 (1:1000, caspase-3), rabbit anti-cytochrome c (1:500), rabbit anti-Bax (1:1000), and rabbit anti-Bcl-2 (1:1000). The protein load of each channel was detected using an anti-GAPDH antibody (1:5000) and beta-actin antibody (1:2000). Goat anti-rabbit or anti-mouse secondary antibodies (1:12,000) were used before chemiluminescent detection. Immunoblots were visualized using BeyoECL Plus. Results were expressed as a percentage of control.

### ELISA analysis

We collected serum in coagulation tubes and centrifuge (3000 rpm, 15 min), and Collected the plasma and stored it at − 80 °C. Determination of serum osteocalcin (OCN) (Meimian Biotechnology, Yancheng, Jiangsu, China), Tartrated Resistant Acid Phosphatse (TRAP) (Meimian Biotechnology, Yancheng, Jiangsu, China), C-terminal telopeptide II (CTX-II) (Meimian Biotechnology, Yancheng, Jiangsu, China), A Lkaline Phosphatase (ALP) (Meimian Biotechnology, Yancheng, Jiangsu, China), Procollagen I Intact N-Terminal (PINP) (Meimian Biotechnology, Yancheng, Jiangsu, China), Interleukin-6 (IL-6) (Meimian Biotechnology, Yancheng, Jiangsu, China), C-reactive protein (CRP) (Meimian Biotechnology, Yancheng, Jiangsu, China), tumor necrosis factor -α (TNF-α) (Meimian Biotechnology, Yancheng, Jiangsu, China), and Interleukin-1β (IL-1β) (Meimian Biotechnology, Yancheng, Jiangsu, China) were performed using commercial enzyme-linked immunosorbent assay (ELISA) kits.

### Osteoblast cells experiment

Preosteoblast MC3T3-E1 cells were cultured overnight in 48-well plates and treated with 50 g/ml ascorbic acid and 10 mM α-glycerophosphate cells for 3 weeks with or without KuA (5, 10, and 20 M). Cells were fixed with cold 70% ethanol for 10 min at room temperature, then rinsed with water. Calcium precipitation in mineralized cells was determined by staining with Alizarin Red S (Sigma-Aldrich). Alizarin S red staining was positive under light microscopy. To quantify this, cells were extracted with 10% cetylpyridinium chloride for 1 h and seeded into 96-well plates. We measured the absorbance of the extract at 550 nm (BIO-RAD; Hercules, CA, USA) (Park et al. 2019b).

### RT-PCR analysis and oxidative stress

Muscle and connective tissue of the distal left tibia were washed, frozen in liquid nitrogen, and stored at − 80 °C. Frozen tibiae were sprayed with a Bessman tissue sprayer under liquid nitrogen (Spectrum Laboratories, Rancho Dominguez, CA, USA). Total RNA was extracted using Trizol reagent (Invitrogen, Carlsbad, CA, USA). The expression levels of bone metabolism and inflammation-related genes, including *OCN, RANKL, OPG, IL-6*, and *Osterix* (Additional file 1: Table S1). The relative change in gene expression was analyzed by the 2-ΔΔCT method.

The levels of MDA, H2O2, cytochrome, and the activities of MnSOD and CuZnSOD were measured using commercially available kits (CAK) according to the manufacturer’s instructions.

### Transient transfections of siRNA molecules

The transient transfection of siRNA molecules *PYGM* (Life Technologies) was performed using RNAiMAX reagent as instructed by the manufacturer (Life Technologies). Briefly, MC3T3-E1 cells were transfected into 6-well plates containing 10 nM siRNA molecule or RNAiMAX shuffling control. Cells were then maintained in 2% horse serum, differentiated for 3 days, and 1 ml Tri reagent was collected from three RNA extraction wells or 1 ml RIPA buffer (Thermo Scientific) (containing Halt Protease and Phosphatase Inhibitor Cocktail; Thermo Scientific) for RT-PCR analysis (Myers et al. 2013).

### Bioactive ingredients and target genes of LC

Traditional Chinese Medicine Systems Pharmacology Database and Analysis Platform (TCMSP, https://tcmspw.com/index.php) was used to analyze the ingredients of LC such as oral bioavailability (OB), drug-likeness (DL), intestinal epithelial permeability, blood–brain barrier penetrability, and water solubility. After screening the TCMSP dataset, 22 bioactive ingredients were obtained (Ru et al. 2014). In preparation for molecular adaptation, constituent SDF files of 13 active molecules (http://pubchem.ncbi.nlm.nih.gov) were downloaded from the PubChem database after calculation in Chemdraw 3D Ultra software.

### Differential Genes analysis of OP

The genetic samples (GSM1369766) of patients with low BMD and high BMD were obtained from the GEO dataset. The ‘limma’ package was installed in Perl and the sample values were patched and converted to log2 (logFC). Samples with P-value < 0.005 and ∣log2 fold change∣ > 1 were considered to have statistically significant and selected as differential genes. Then we created a volcano gene map from the sample and selected the top 20 most important up-down corrected genes for the heatmap.

### Protein–Protein Interaction (PPI) Network

In a PPI network, the concentration (DC) of each node is the number of edges per node. The higher the degree, the higher the center position of the node. The relay center (BC) receives the location of the node among other nodes. Specifically, it is the ratio of the number of shortest paths through this node to the total number of shortest paths in the network. DC and BC reflect the influence of individual nodes on the entire network. They describe topological centrality in terms of network connectivity and controllability.

The ‘biogenetic, cytoNAC’ package was installed in Cytoscape 3.8.0 and was used to enter the crossover gene and select the ‘Homo sapiens’ parameter. Data for constructing the PPI network were sourced from six main experimental research databases: Human Protein Reference Database, Biological General Repository for Interaction Datasets, Database of Interacting Proteins, IntAct molecular interaction database, Molecular INTeraction Database, and Biomolecular Interaction Network Database. We selected this method “input nodes and its neighbors” to obtain a PPI network and performed a topology analysis based on the central location of the network.

### LC-OP-Potential target genes network

LC-related target genes were selected from the TCMSP database based on chemical similarity and pharmacophore models. We calibrated LC-related target names to default names using the UniProt database (https://www.uniprot.org/) (Szklarczyk et al. 2016). The TCMSP formula was adapted using Cytoscape web page generation software.

### GO and KEGG enrichment

Go enrichment analysis examines gene function at three levels: biological process (BP), cellular component (CC), and molecular function (MF). BP mainly involves aspects of response to a steroid hormone, response to oxygen levels, and regulation of lipid metabolic process. CC is mainly related to the integral component of the postsynaptic membrane, GABA receptor complex, and GABA-A receptor complex. MF is remarkably linked with neurotransmitter receptor activity, steroid hormone receptor activity, and GABA-A receptor activity.

Firstly, we changed the names of the potential target genes from R-package (org.Hs.eg.db, version 3.8) to entrezID which helps to exclude errors caused by capitalization or abbreviations of the target names. Then, we used the R-package ‘DOSE’, ‘cluster profile’, and ‘pathview’ to visualize the biological functions of graphene oxide and analyze the enrichment of the KEGG pathway for which the p-value was < 0.05 for further analysis.

### Molecular Docking analysis

We used CB-Dock Internet Molecular Docking Technology to select active components of potential LC-OP target gene networks and Docked with *PYGM* receptors (http://cao.labshare.cn/cbdock//) (Liu et al. 2020). *PYGM* (protein ID is 6y5o) and the active ingredients were uploaded to the CB-Dock website. After determining the coordinates of the docking pocket, molecular docking and conformational assessment were performed using the CB docking station. The lower the VINA score, the more stable the ligand binding. Finally, receptors are screened for the binding activity of compounds to the target.

### Statistical analysis

All measurements were presented as the mean ± standard deviation (SD) and a P-value of ≤ 0.05 was considered statistically significant. The bodyweight of the time-course study was analyzed by two-way repeated-measures analysis of variance (ANOVA). Data were analyzed for the main effect and timing of the intervention. One-way ANOVA followed by Tukey multiple analysis was performed using GraphPad Prism 8.02 (La Jolla, CA, USA).

## Results

### KuA administration protects against OVX-induced bone loss

**Fig. 1:**
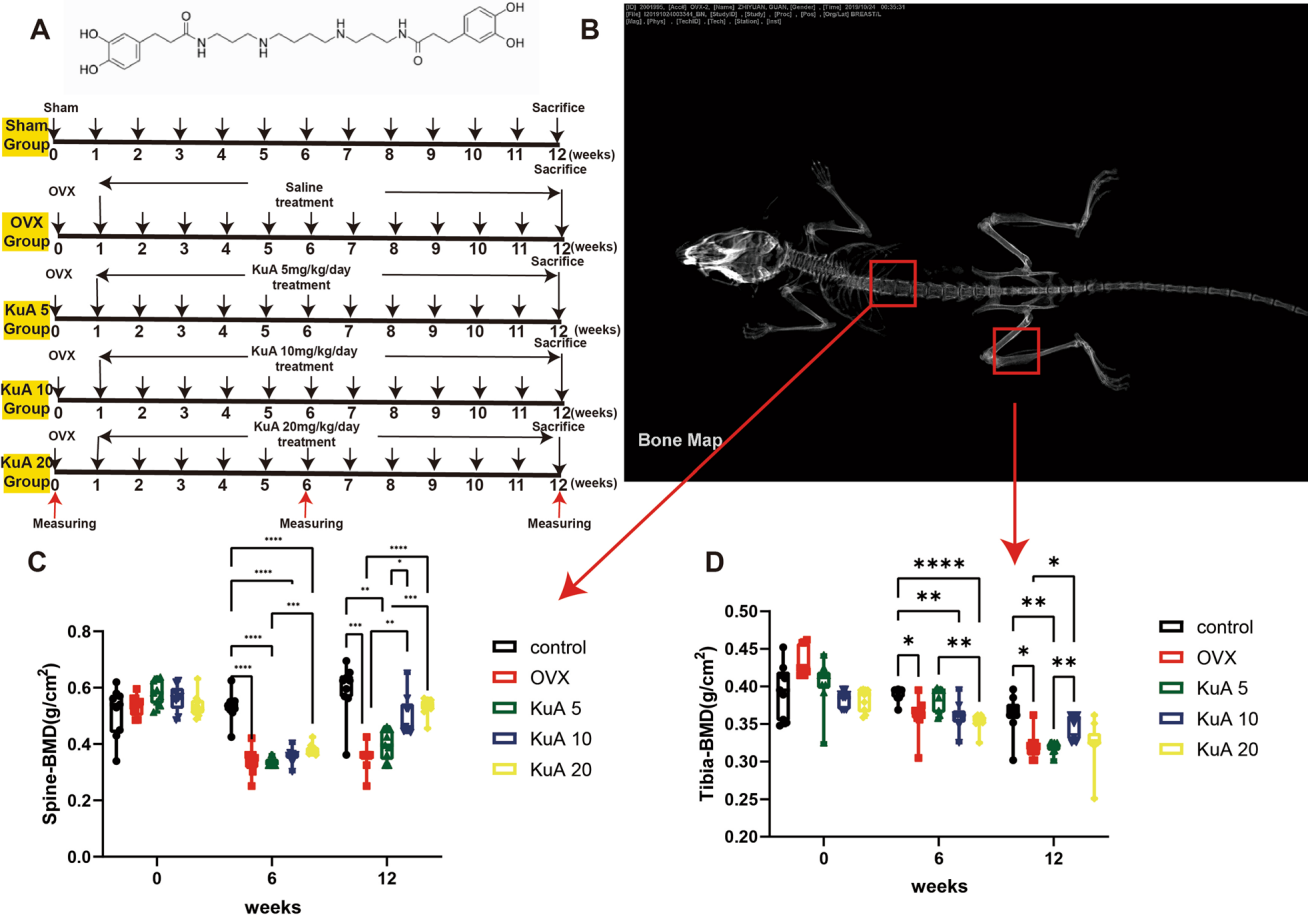
The experimental result show that KuA significantly increased the bone mineral density of the spine and tibia in OVX mice. **A** KuA spectrometry analyses and total experiment flowchart. **B** Representative figure of bone mineral density in mice. **C** BMD of L4. **D** BMD of the tibia. These results found that KuA improves the bone mass of the tibia and spine in ovariectomized mice. *P < 0.05, **P < 0.01, ***P < 0.001, ****P < 0.0001. KuA: Kukoamine A; OVX: ovariotomy; L4: Lumber 4; BMD: bone mineral density

KuA isolated from LC extracts was identified by magnetic resonance imaging (NMR) and mass spectrometry (Fig. 1A). As expected, the OVX mice showed a significant reduction of BMD in lumber 4 and right tibia at 6 weeks and 12 weeks after surgery. KuA 10 mg and 20 mg administration inhibited the reduction of BMD at 12 weeks (Fig. 1B, C, Additional file 1: Figure S2).

**Fig. 2:**
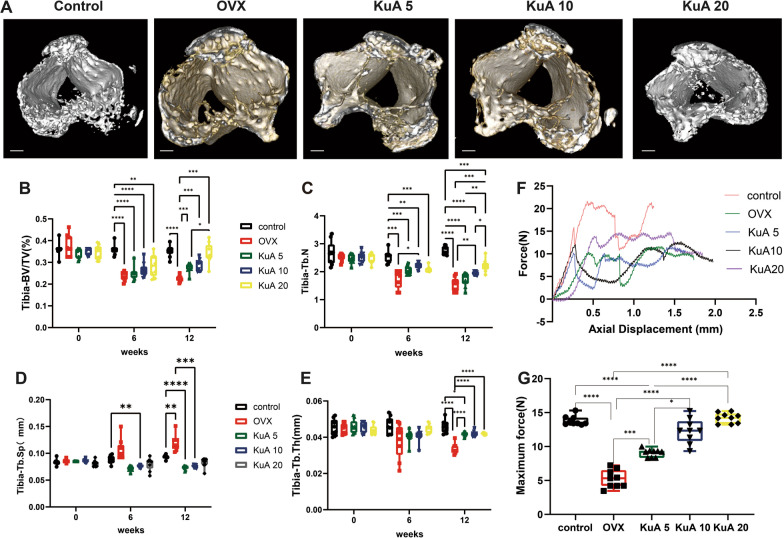
KuA significantly improved the tibia bone microstructure and mechanical properties in OVX mice. **A** Representative figure in the tibia. **B** BV/TV of the tibia. **C** Tb.N of tibia. **D** Tb.Sp of the tibia. **E** Tb.Th of the tibia. These results found that KUA improved the bone microstructure of the tibia in ovariectomized mice. **F**, **G** The maximum force of the tibia. Mechanical results showed that KUA improve the maximum stress of the tibia in ovariectomized mice. The bar is 0.7 mm. *P < 0.05, **P < 0.01,***P < 0.001,****P < 0.0001. KuA: Kukoamine A; OVX: ovariotomy; L4: Lumber 4; BMD: bone mineral density. BV/TV: bone volume over total volume; Tb.Th: trabecular thickness; Tb.N: trabecular number, Tb.Sp: trabecular spacing

The bone microarchitecture of the right tibia analysis revealed that KuA administration with 5 mg, 10 mg, and 20 mg prevented the tibia-BV/TV than the OVX group at 12 weeks after treatment. Tibia-Tb.N also showed a remarkable improvement by KuA 10 mg compared with the OVX group at 6 weeks and 12 weeks. However, at 12 weeks after treatment, KuA 10 mg can decrease Tibia-Tb.Sp significantly more than the OVX group. In addition, KuA 5 mg, 10 mg, and 20 mg can also increase tibia-Tb.Th significantly than the OVX group 12 weeks after treatment (Fig. 2A–E, Additional file 1: Figure S3).

**Fig. 3:**
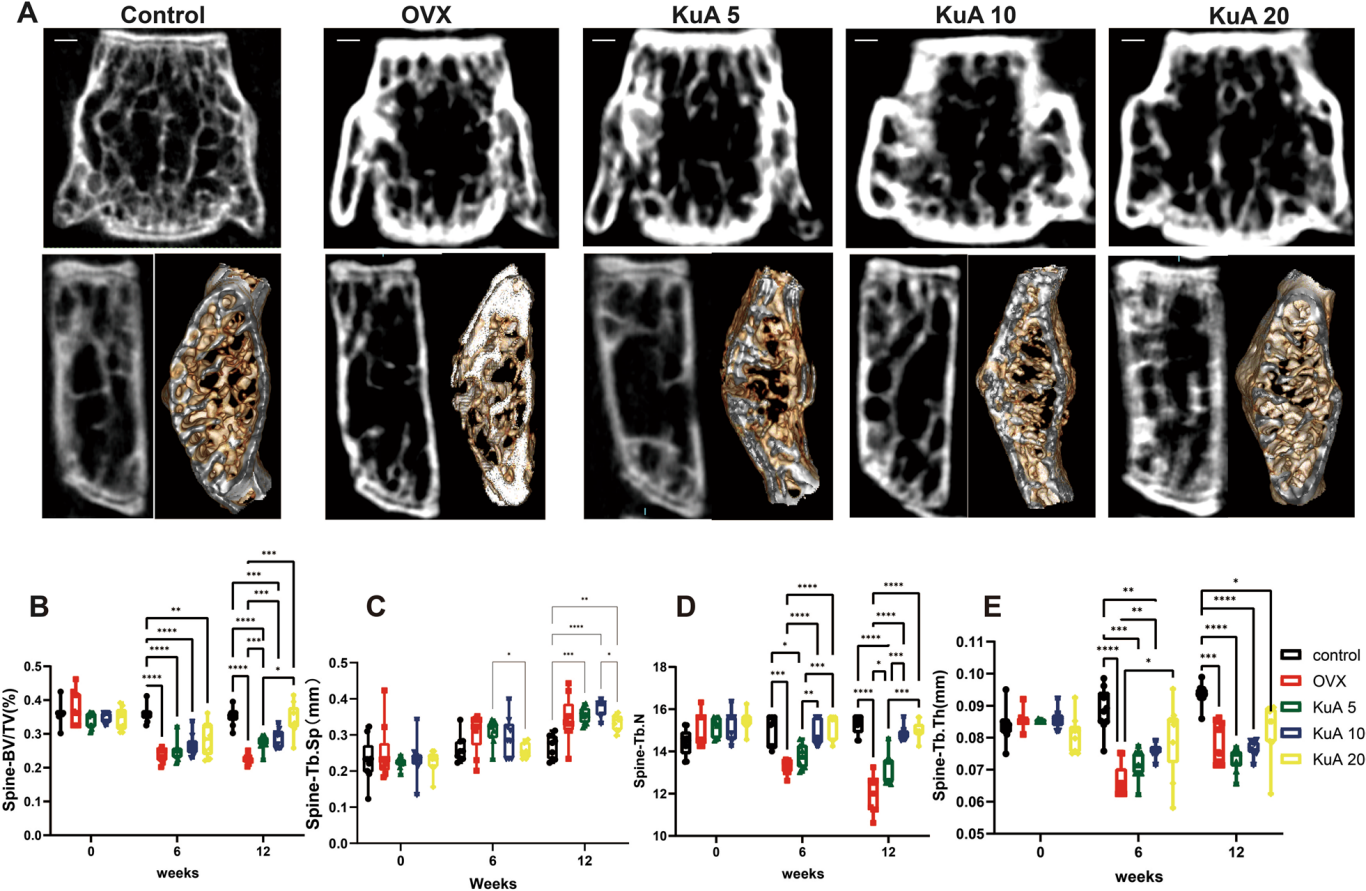
KuA significantly increased the bone microstructure of the spine in OVX mice. **A** Representative figure in L4. **B** BV/TV of L4. **C** Tb.Sp of L 4. **D** Tb.N of L 4. **E** Tb.Th of L4. These results found that KuA improved the bone microstructure of the spine in ovariectomized mice. The bar is 0.3 mm. *P < 0.05, **P < 0.01,***P < 0.001,****P < 0.0001. KuA: Kukoamine A; OVX: ovariotomy; L4: Lumber 4; BMD: bone mineral density. BV/TV: bone volume over total volume; Tb.Th: trabecular thickness; Tb.N: trabecular number, Tb.Sp: trabecular spacing

Lumber 4 microarchitecture was also shown in Fig. 3A. For spine Tb.N and BV/TV, KuA 5 mg, 10 mg, and 20 mg improved significantly than the OVX group at 12 weeks, but for spine Tb.Sp and Tb.Th, KuA showed no significant difference in the OVX group at 12 weeks after treatment (Fig. 3B–E).

As shown in Table 1, there was a significant reduction in Ct.Th, Tt.Ar and Ct.Ar/Tt.Ar in the OVX group compared to the control. After KuA intervention, Ct.Th improved significantly in the KuA 20 group than the OVX group. The KuA 10 and KuA 20 groups improved significantly relative to the OVX group in Ct.Ar/Tt.Ar.

**Table 1:** Changes in serological markers and cortical bone indicators in KuA-treatment osteoporotic mice

Characteristics	Groups	Control	OVX	KuA5	KuA10	KuA20
Bone turnover biomarkers	OCN (ng/ml)	3.22 ± 0.25*^&	1.16 ± 0.15	1.65 ± 0.21**#	2.36 ± 0.24**#^	3.62 ± 0.28**^&
	TRAP (ng/ml)	10.62 ± 0.698*	12.64 ± 1.59	11.96 ± 1.85#	10.12 ± 2.61*	10.06 ± 1.82*
	CTX-II (ng/ml)	0.081 ± 0.001*	0.086 ± 0.002	0.085 ± 0.003	0.084 ± 0.001	0.084 ± 0.002*
	ALP (ng/ml)	1.26 ± 0.26*^&	0.88 ± 0.14	0.95 ± 0.08#	0.99 ± 0.21#	1.11 ± 0.19*^
	PINP (ng/ml)	0.86 ± 0.11*	0.61 ± 0.09	0.67 ± 0.03#	0.72 ± 0.12	0.78 ± 0.15^
Cortical bone indicators	Ct.Th (mm)	0.14 ± 0.06*	0.11 ± 0.03	0.12 ± 0.05#	0.12 ± 0.04#	0.13 ± 0.02*
	Tt.Ar (mm^2^)	1.35 ± 0.36*	1.62 ± 0.15	1.58 ± 0.23	1.54 ± 0.13	1.42 ± 0.42*^
	Ct.Ar (mm^2^)	0.68 ± 0.03	0.72 ± 0.08	0.69 ± 0.04	0.69 ± 0.07	0.68 ± 0.05
	Ct.Ar/Tt.Ar (%)	62.88 ± 11.62*	42.69 ± 12.95	51.67 ± 12.86#	58.61 ± 14.62*	62.59 ± 21.61*^&

#compare with the control group. * compare with OVX group. ^ compare with KA5 group. & compare with KA10 group.Tt.Ar, the total cross‐sectional area inside the periosteal envelope; Ct.Ar, cortical bone area; Ct.Th, average cortical thickness; Ct.Ar/Tt.Ar, cortical area fraction; KuA5, OVX + 5 mg/kg/day of KuA; KuA10, OVX + 10 mg/kg/day of KuA; KuA20, OVX + 20 mg/kg/day of KuA; OCN, osteocalcin; ALP, Alkaline Phosphatase; TRAP: Triiodothyronine Receptor Auxiliary Protein; CTX-II: Collagen Type II Alpha 1 Chain; PINP: Procollagen I N-Terminal Propeptide; Tt.Ar: total cross‐sectional area inside the periosteal envelope; Ct.Ar: cortical bone area; Ma.Ar: bone marrow area; Ct.Th: average cortical thickness; Ct.Ar/Tt.Ar:cortical area fraction

For bone turnover biomarkers, KuA treatment can improve the osteogenesis-related indicators including serum OCN and ALP but decrease osteoclast-related indicators TRAP and CTX-II compared with the OVX group (Table 1).

Biomechanical analysis of the right tibia at 12 weeks after treatment was shown in Fig. 6F, KuA 5 mg, 10 mg, and 20 mg administration can significantly improve the maximum force, stiffness, and displacement of than OVX group (Fig. 6G, Additional file 1: Figure S4A, B). however, only KuA 5 mg administration can improve the energy absorption of than OVX group (Additional file 1: Figure S4).

### Identification of KuA as an osteogenic suppressor

LC contains 22 active ingredients totally in the TCMSP dataset. The target genes were then screened from these active ingredients of LC by DrugBank dataset as the conditions. There were 321 target genes obtained for LC active ingredients. The removal of duplicates after verification yielded 242 target genes (Table 2).

**Table 2:** The total available compounds of Lycium chinense Mill

Index	Mol ID	Molecule name	MW	AlogP	Hdon	Hacc	OB (%)	Caco-2	BBB	DL	FASA −	HL
9	MOL002224	Aurantiamide acetate	444.57	4.526	2	6	58.38079	0.40909	− 0.22162	0.5883	84.5	7.035985
5	MOL002218	Scopolin	354.34	− 0.288	4	9	56.44689	− 1.05198	− 1.75118	0.38714	138.82	3.006583
1	MOL001552	OIN	289.41	1.721	1	4	45.97058	0.43143	0.09193	0.19317	49.77	4.469495
11	MOL002228	Kulactone	452.74	6.233	0	3	45.43808	0.85591	0.1607	0.81578	43.37	5.521435
16	MOL000449	Stigmasterol	412.77	7.64	1	1	43.82985	1.44458	1.00045	0.75665	20.23	5.574595
2	MOL001645	Linoleyl acetate	308.56	6.847	0	2	42.10077	1.35826	1.08413	0.19845	26.3	7.478521
7	MOL002221	Kukoamine A	530.74	1.78	8	10	42.0846	− 0.20726	− 2.11576	0.56398	163.18	0
4	MOL001790	Linarin	592.6	− 0.178	7	14	39.84373	− 1.68135	− 2.76521	0.70925	217.97	16.06778
22	MOL000953	CLR	386.73	7.376	1	1	37.8739	1.43101	1.12678	0.67677	20.23	4.518834
14	MOL000296	Hederagenin	414.79	8.084	1	1	36.91391	1.31876	0.96428	0.75072	20.23	5.347511
15	MOL000358	Beta-sitosterol	414.79	8.084	1	1	36.91391	1.32463	0.98588	0.75123	20.23	5.355491
8	MOL002222	Sugiol	300.48	4.987	1	2	36.11353	1.14054	0.69922	0.27648	37.3	14.61994
3	MOL001689	Acacetin	284.28	2.585	2	5	34.97357	0.67146	− 0.04689	0.24082	79.9	17.24847
6	MOL002219	Atropine	289.41	1.996	2	4	34.52789	0.15341	− 0.29661	0.21417	60.77	3.117746
18	MOL000472	Emodin	270.25	2.492	3	5	24.39832	0.22289	− 0.66096	0.23916	94.83	0
20	MOL000008	Apigenin	270.25	2.334	3	5	23.06216	0.4256	− 0.6109	0.21306	90.9	0
19	MOL000476	Physcion	284.28	2.743	2	5	22.2864	0.52191	− 0.40229	0.26659	83.83	0
13	MOL000295	Alexandrin	576.95	6.337	4	6	20.63194	− 0.1993	− 0.80971	0.62697	99.38	0
10	MOL002226	Lyciumin A	874.02	− 0.238	10	20	10.07929	− 2.51813	− 2.66337	0.1977	306.7	0
21	MOL000880	Tricosane	324.71	10.864	0	0	8.33048	1.84718	1.6799	0.2088	0	0
12	MOL002229	HEPTACOSANE	380.83	12.689	0	0	8.18071	1.87942	1.80044	0.36155	0	0
17	MOL000458	Campesterol	400.76	7.972	1	1	5.568613	1.59665	1.40847	0.71577	20.23	0

**Fig. 4:**
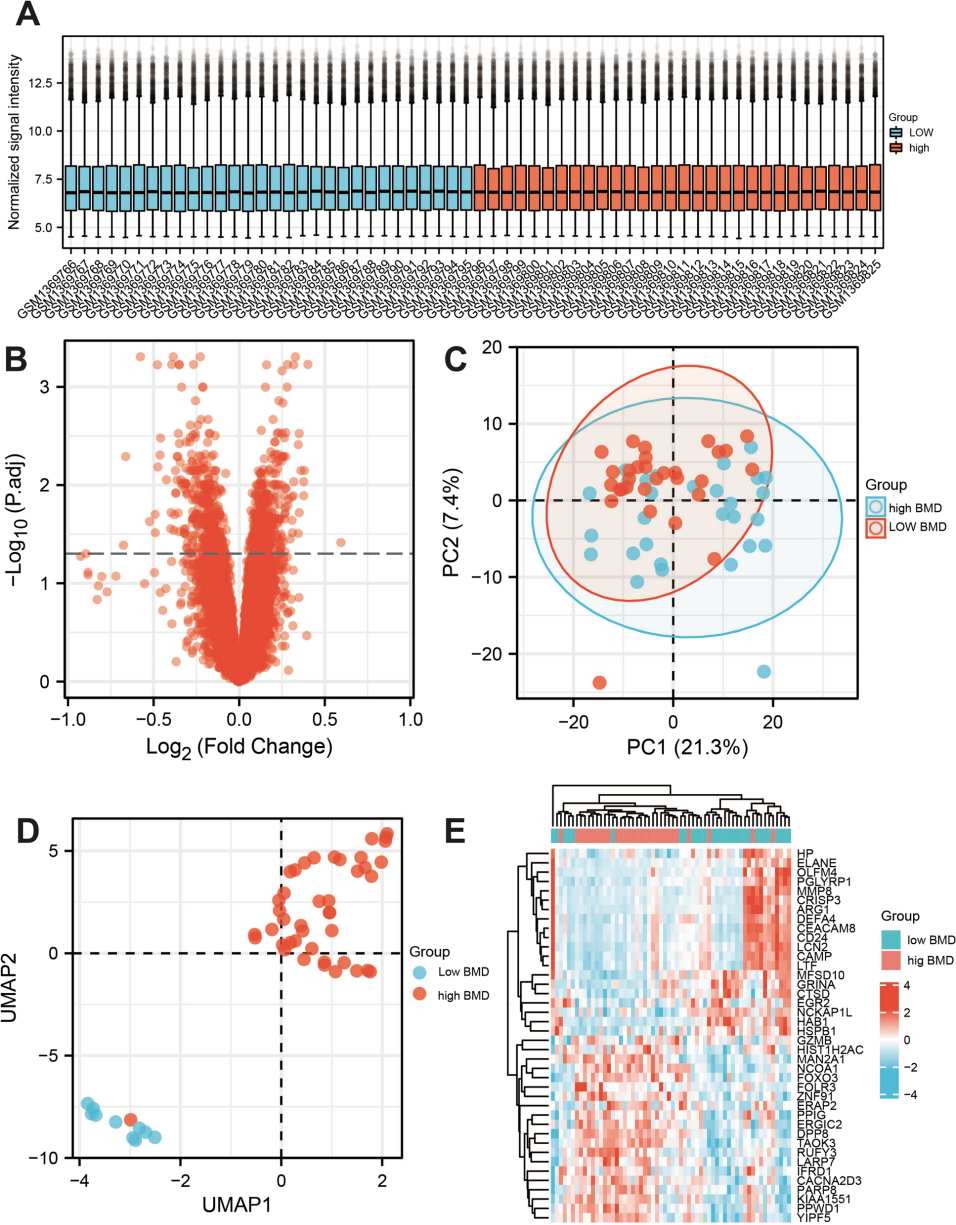
The gene expression analyses in osteoporosis samples from the GEO dataset. **A** Gene normalization diagram shows this dataset has a good consistency. **B** Gene volcano map. **C** Principal genetic analysis. **D** Uniform Manifold Approximation and Projection. **D** Heatmap analysis found that the top 20 genes expressed in GEO dataset. UMAP: Uniform Manifold Approximation and Projection, PCA: Principal genetic analysis

**Fig. 5:**
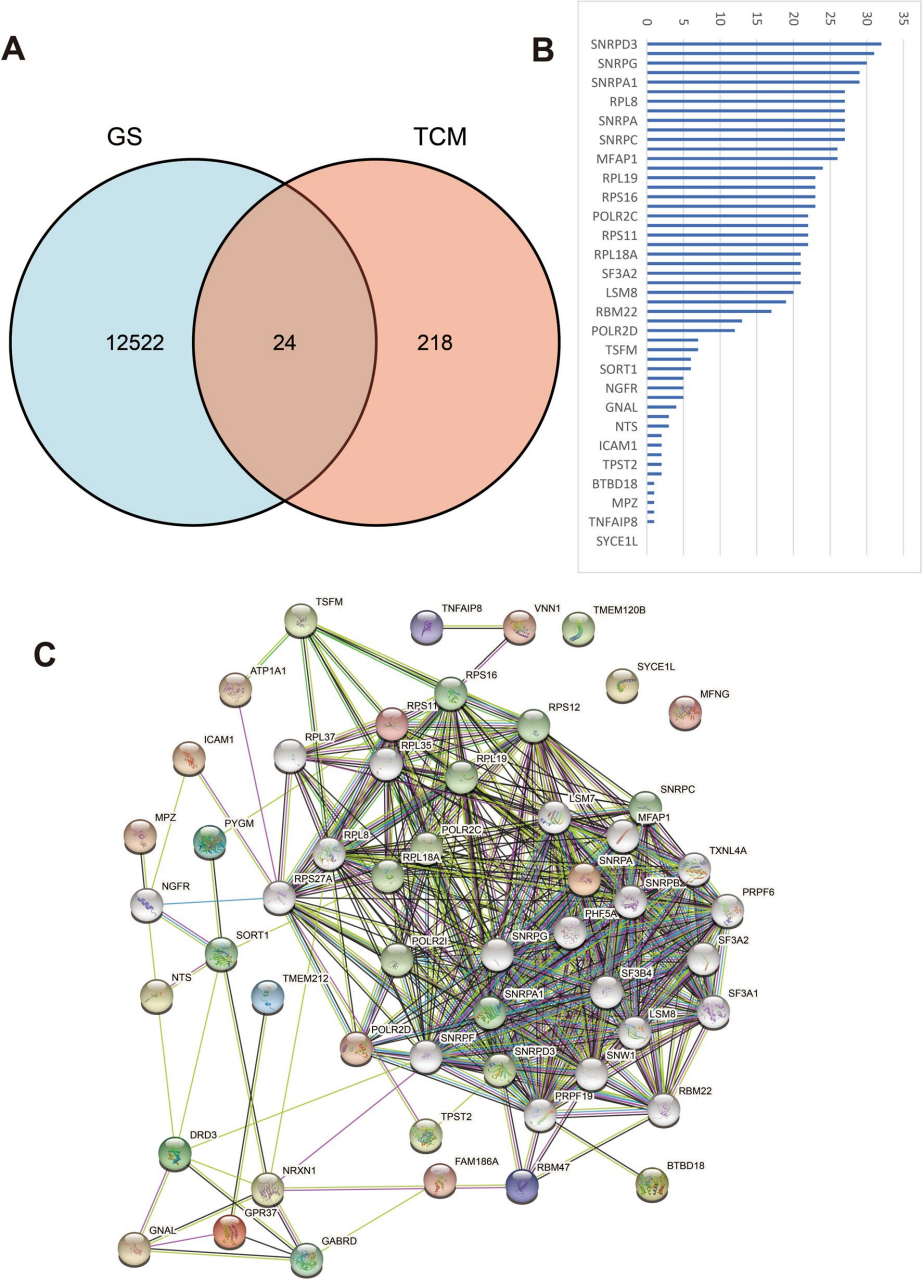
Potential target genes and PPI network map of KuA for OP. **A** The Venn results of potential genes of KuA therapy for OP. **B** Counts and lists of the top genes of PPI network map. **C** The PPI network map of 24 target genes. 723 protein nodes and 8743 edges were obtained for intersection genes. After screening with DC > 61 and a BC range of 20–113.2, the first 20 proteins were selected in Table 5 (in descending order of degree), with a total of 322 edges. PPI: Protein–Protein Interaction; KuA: Kukoamine A; OP: osteoporosis

By comparing 30 low BMD samples and 30 high BMD samples in the GEO database. A total of 12,548 differential genes were required, including 7167 up-regulated genes and 5381 down-regulated genes. After screening with a P-value < 0.005 and ∣log2 (fold change)∣ > 1, the gene volcano map was analyzed in Fig. 4A, B. PCA analysis, UMAP, and heatmap analysis were shown in Fig. 1C–E. The differential genes in the disease samples were normally distributed, and the number of up-regulated genes was greater than the number of down-regulated genes. Table 3 listed the 20 most important up-and-down-regulated genes. Using Venny 2.1 software to hybridize OP target genes and LC target genes, 24 potential target genes were obtained, as shown in Fig. 5A. We found that *PYGM* has the highest LogFC value (Table 4).

**Table 3:** The top 20 genes are upregulated and downregulated

Gene names	LogFC	P value	Regulation direction
*LCN2*	− 0.92606	0.003277	Down
*CAMP*	− 0.89523	0.00297	Down
*OLFM4*	− 0.88312	0.007425	Down
*DEFA4*	− 0.88296	0.00825	Down
*CEACAM8*	− 0.82947	0.024539	Down
*LTF*	− 0.8221	0.013364	Down
*CRISP3*	− 0.80266	0.008933	Down
*CD24*	− 0.77216	0.017416	Down
*MMP8*	− 0.72018	0.008758	Down
*ELANE*	− 0.67617	0.001989	Down
*HAB1*	− 0.66318	2.75E−05	Down
*NCKAP1L*	− 0.57676	1.55E−07	Down
*EGR2*	− 0.55255	0.012038	Down
*HP*	− 0.5232	0.000704	Down
*ARG1*	− 0.4947	0.007518	Down
*PGLYRP1*	− 0.49116	0.000471	Down
*GRINA*	− 0.49006	0.001152	Down
*MFSD10*	− 0.4782	6.16E−07	Down
*HSPB1*	− 0.4754	4.22E−05	Down
*CTSD*	− 0.46251	2.28E−05	Down
*YIPF5*	0.308376	0.00068	Up
*PARP8*	0.310742	0.001162	Up
*HIST1H2AC*	0.313757	0.077778	Up
*IFRD1*	0.315896	0.006762	Up
*ERGIC2*	0.316251	0.000205	Up
*FOXO3*	0.318552	5.93E−07	Up
*LARP7*	0.319056	8.96E−05	Up
*NCOA1*	0.327933	5.32E−08	Up
*KIAA1551*	0.345275	0.001193	Up
*MAN2A1*	0.345422	1.54E−05	Up
*GZMB*	0.349538	0.022463	Up
*TAOK3*	0.35036	8.64E−06	Up
*RUFY3*	0.350575	0.000429	Up
*PPWD1*	0.360551	5.56E−05	Up
*PPIG*	0.376994	0.000203	Up
*CACNA2D3*	0.377052	8.98E−05	Up
*ZNF91*	0.380684	5.14E−05	Up
*ERAP2*	0.394709	0.11964	Up
*DPP8*	0.401369	3.81E−07	Up
*FOLR3*	0.59427	0.001779	Up

**Table 4:** The 20 intersection genes sorted by logFC

Intersection gene	LogFC	P value
*BTBD18*	− 0.000555667	0.983394254
*DRD3*	0.002213333	0.932333004
*SYCE1L*	0.002595333	0.885291809
*ATP1A1*	0.010409333	0.839478969
*POLR2D*	− 0.010996333	0.746612769
*ICAM1*	− 0.020975667	0.696896549
*TNFAIP8*	− 0.041541333	0.569759794
*RBM47*	− 0.045589667	0.455734347
*VNN1*	0.193443667	0.337112369
*TSFM*	0.047431667	0.256808881
*NRXN1*	0.039595	0.148943914
*FAM186A*	0.040678	0.059556681
*MFNG*	− 0.109743	0.051251272
TMEM212	− 0.052631333	0.040565698
TPST2	− 0.154919333	0.030568761
RPS11	− 0.095648333	0.014220426
GABRD	− 0.096749333	0.012048201
NTS	− 0.08813	0.008618444
TMEM120B	− 0.080779	0.005985507
PYGM	0.102682333	0.000930636

### PPI network and topological analysis

The combination of DC and BC values is an effective method for reliable monitoring of important proteins (Wang et al. 2013). As shown in Fig. 2, 723 protein nodes and 8743 edges were obtained for intersection genes. After screening with DC > 61 and a BC range of 20–113.2, the first 20 proteins were selected in Table 5 (in descending order of degree), with a total of 322 edges. Among the 20 proteins, five proteins were predicted targets of the active ingredients, with their corresponding genes including *NTRK1, MCM2, CUL3, NPM1*, and *FN1* (Fig. 5B, C, Table 5).

**Table 5:** Topological analysis results by degree—the first 20 proteins

Gene names	Annotation	Closeness centrality	Degree
*NTRK1*	Neurotrophic receptor tyrosine kinase 1	0.582762	218
*MCM2*	Minichromosome maintenance complex component 2	0.574324	210
*CUL3*	Cullin 3	0.557638	197
*NPM1*	Nucleophosmin	0.555556	181
*FN1*	Fibronectin 1	0.555037	188
*HNRNPU*	Heterogeneous nuclear ribonucleoprotein U	0.5494	170
*ESR1*	Estrogen receptor 1	0.537975	167
*CDK2*	Cyclin dependent kinase 2	0.537975	154
*RPS11*	Ribosomal protein S11	0.534591	171
*RPS3A*	Ribosomal protein S3A	0.534111	148
*ITGA4*	Integrin subunit alpha 4	0.533632	164
*RPS3*	Ribosomal protein S3	0.533632	150
*CAND1*	Cullin associated and neddylation Dissociated 1	0.533154	165
*RPS4X*	Ribosomal protein S4, X-linked	0.530303	144
*COPS5*	COP9 signalosome subunit 5	0.524691	148
*RPS14*	Ribosomal protein S14	0.524691	146
*RPS16*	Ribosomal protein S16	0.524229	143
*VCAM1*	Vascular cell adhesion molecule 1	0.523307	146
*CUL1*	Cullin 1	0.519197	153
*ICAM1*	Intercellular adhesion molecule 1	0.510292	161

### Construction and analysis of the LC-OP-potential Target gene network

**Fig. 6:**
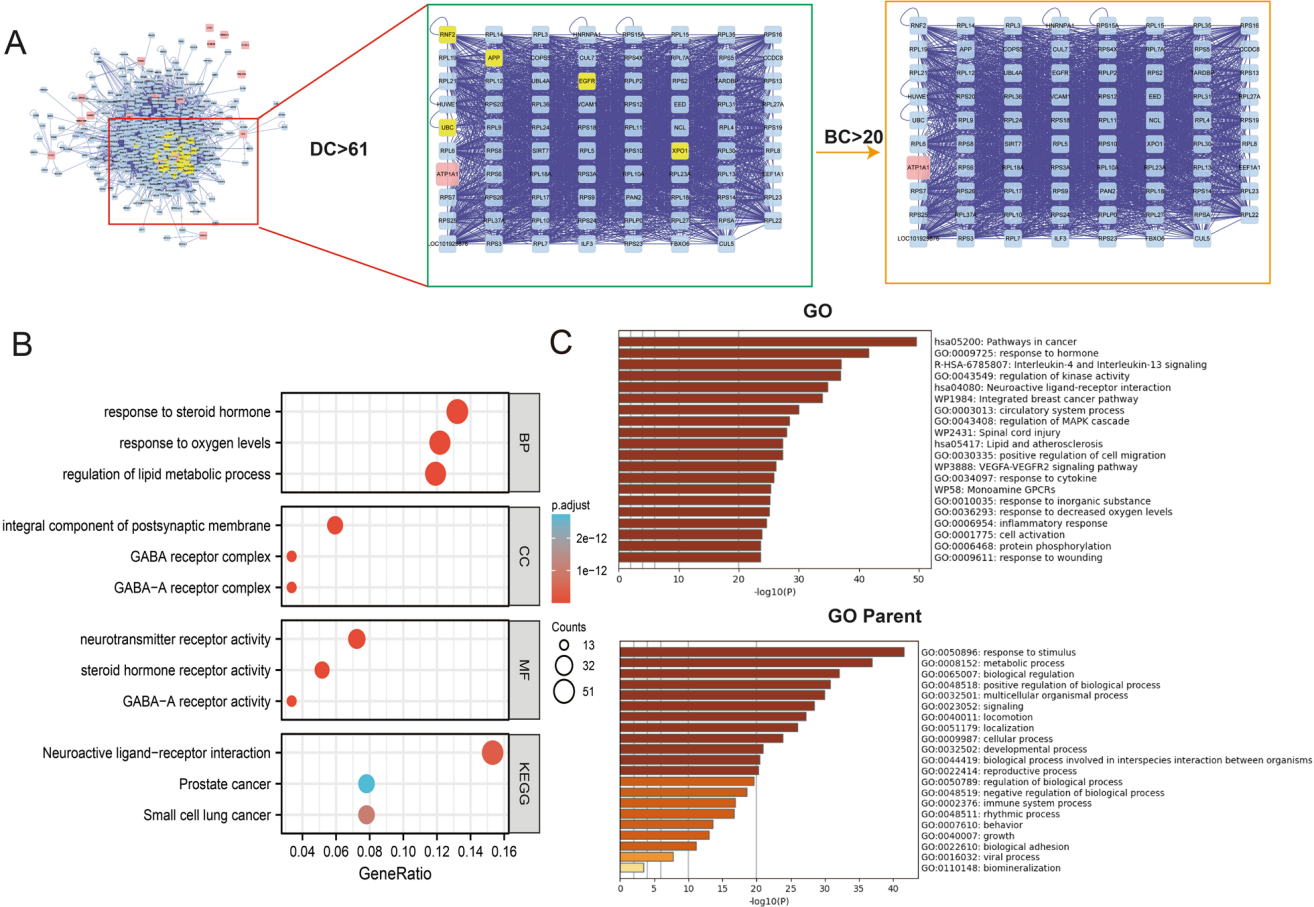
Topological analysis of the protein–protein interaction network (**A**) and GO/KEGG enrichment analysis (**B**, **C**). GO: genetic selection; KEGG: biological pathways. GO patents were linked with response to stimulus, metabolic process, and biological regulation

The gene and miRNA prediction network is an important method to predict miRNA and thus has certain significance to analyze the relationship between LC-OP (Fig. 6). The LC-OP-potential Target gene network was constructed by Cytoscape software (version 3.7.1). From the network of potential LC-OP target genes, a total of 42 nodes and 266 lines were derived, and coumarin A had the highest level in the process, which also explains its important role in the network (Bai et al. 2021).

### GO and KEGG enrichment analysis

According to the KEGG enrichment results, the mechanism of active ingredients of LC in the treatment of OP mainly focuses on the interaction of neuroactive ligands with receptors, postoperative cancer, and small cell lung cancer (Fig. 6B).

GO enrichment analysis also included heatmap-related select GO patients, GO cell-type signatures, GO disease, GO trust, GO paGenbase and GO transcription factor. Heatmap-related select GO patients were associated with the pathway in cancer and response to the hormone. GO patents were linked with response to stimulus, metabolic process, and biological regulation. SP1, RELA, and NFKB1 pathways also played a key role in GO TRRUST (Bai et al. 2021) (Fig. 6C, Additional file 1: Figure S5). Additionally, the PYGM-related glycogene synthesis and degrade pathway were shown in Additional file 1: Figure S6.

### Molecular Docking

**Fig. 7:**
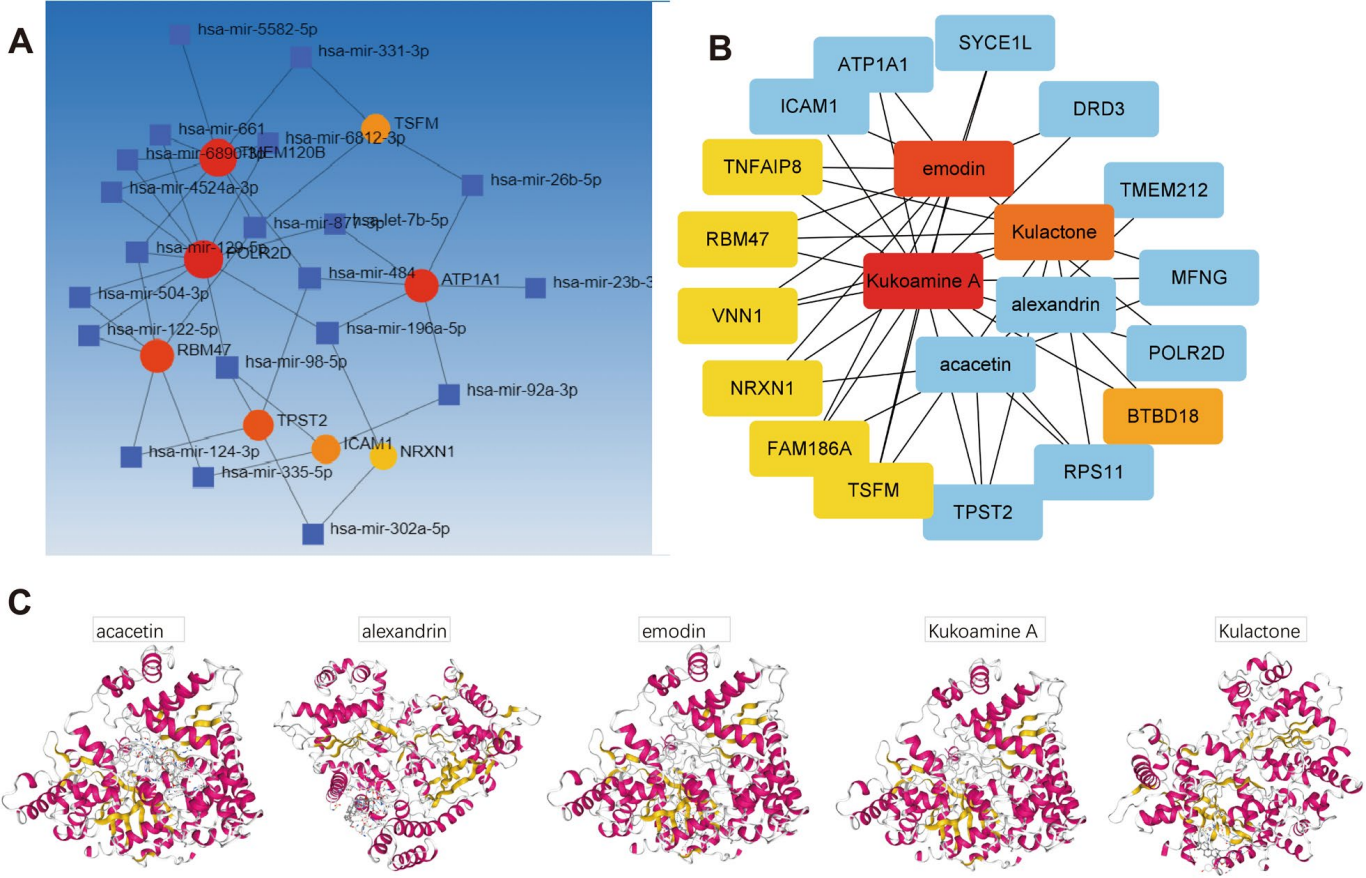
**A** Target genes-miRNA. **B** TCM compound-disease regulatory network. **C** The 3D map of binding of KuA. The Vina score results of KuA, Linarin, aurantiamide acetate, and acacetin increased steadily, indicating that KuA has the strongest and most stable binding affinity for *PYGM*. 3D images of acacetin, alexandrine, emodin, KuA, and Kulactone to *PYGM*. KuA: Kukoamine A; OVX: ovariotomy; TCM: Traditional Chinese Medicine

From the network of potential LC-OP target genes, five active ingredients were selected, namely KuA, Emodin, Kulactone, Alexandrine, and Acacetin (Table 6). A low Vina score indicates a stronger and more stable interaction between the compound and the receptor. Molecular docking between Active ingredients and target genes were shown in Table 7. The Vina score results of KuA, Linarin, aurantiamide acetate, and acacetin increased steadily, indicating that KuA has the strongest and most stable binding affinity for *PYGM*. These results suggest that KuA may be the most suitable starting material for *PYGM*. 3D images of acacetin, alexandrine, emodin, KuA, and Kulactone to *PYGM* were shown in Fig. 7C.

**Table 6:** Molecular docking parameters and results of seven active ingredients in LC binding with PYGM

Molecule name	Vina scores	Cavity size	Center			Size		
			x	y	z	x	y	z
OIN	− 7.8	1837	5	23	29	21	21	27
Kukoamine A	− 12.3	1598	31	24	30	36	36	36
Linarin	− 11.3	1598	31	24	30	24	24	24
Aurantiamide acetate	− 10.1	1598	31	24	30	29	23	23
Acacetin	− 10	1598	31	24	30	29	21	21
Apigenin	− 9.8	1598	31	24	30	29	21	21
Kulactone	− 8.9	1484	26	3	51	24	24	24
Emodin	− 8.9	1598	31	24	30	29	19	19
Scopolin	− 8.6	1598	31	24	30	29	21	21
Physcion	− 8.6	1598	31	24	30	29	20	20
Stigmasterol	− 8.5	1484	26	3	51	25	25	25
Lyciumin A	− 8.4	1484	26	3	51	28	28	28
Hederagenin	− 8.2	1484	26	3	51	25	25	25
Alexandrin	− 7.9	1837	5	23	29	29	29	29
Atropine	− 7.8	1837	5	23	29	21	21	27
Sugiol	− 7.7	1484	26	3	51	26	29	20
Beta-sitosterol	− 7.7	1484	26	3	51	23	29	23
Campesterol	− 7.7	1484	26	3	51	24	24	24
Linoleyl acetate	− 7	1598	31	24	30	29	29	29
HEPTACOSANE	− 7	1598	31	24	30	31	31	31
CLR	− 6.7	1598	31	24	30	36	36	36
Tricosane	− 6.4	1598	31	24	30	28	28	28

**Table 7:** Vina score of LC active components to the target gene molecules

Active components	Genes-Vina score			
	*PYGM*	*TPST2*	*RPS11*	*GABRD*
Acacetin	− 10	− 9	− 7.4	− 7.8
Alexandrin	− 7.9	− 7.6	− 8.4	− 7.7
Apigenin	− 9.8	− 8.2	− 7.3	− 7.7
Atropine	− 7.8	− 8.2	− 6.8	− 7.3
Aurantiamide acetate	− 10.1	− 7.4	− 8.2	− 7.3
Beta-sitosterol	− 7.7	− 7.3	− 7.7	− 7.3
Campesterol	− 7.7	− 7.4	− 7.3	− 7.6
CLR	− 6.7	− 5.7	− 6.2	− 5.9
Emodin	− 8.9	− 9.4	− 8.3	− 8.1
Hederagenin	− 8.2	− 7.6	− 8	− 7.5
HEPTACOSANE	− 7	− 5.4	− 5.6	− 5.5
Kukoamine A	− 12.3	− 7.3	− 6.5	− 6.8
Kulactone	− 8.9	− 7.9	− 8.5	− 9.4
Linarin	− 11.3	− 9	− 9.1	− 8.8
Linoleyl acetate	− 7	− 6.1	− 5.9	− 5.4
Lyciumin A	− 8.4	− 8.5	− 9.8	− 8.7
OIN	− 7.8	− 8.2	− 6.9	− 7.4
Physcion	− 8.6	− 9.6	− 7.5	− 8.3
Scopolin	− 8.6	− 9	− 7.9	− 7.2
Stigmasterol	− 8.5	− 7.4	− 8.1	− 7.3
Sugiol	− 7.7	− 6.8	− 7.4	− 7.5
Tricosane	− 6.4	− 5.3	− 4.6	− 5.4

### KuA increased mRNA expression of osteoblastic differentiation-related genes in OVX mice

**Fig. 8:**
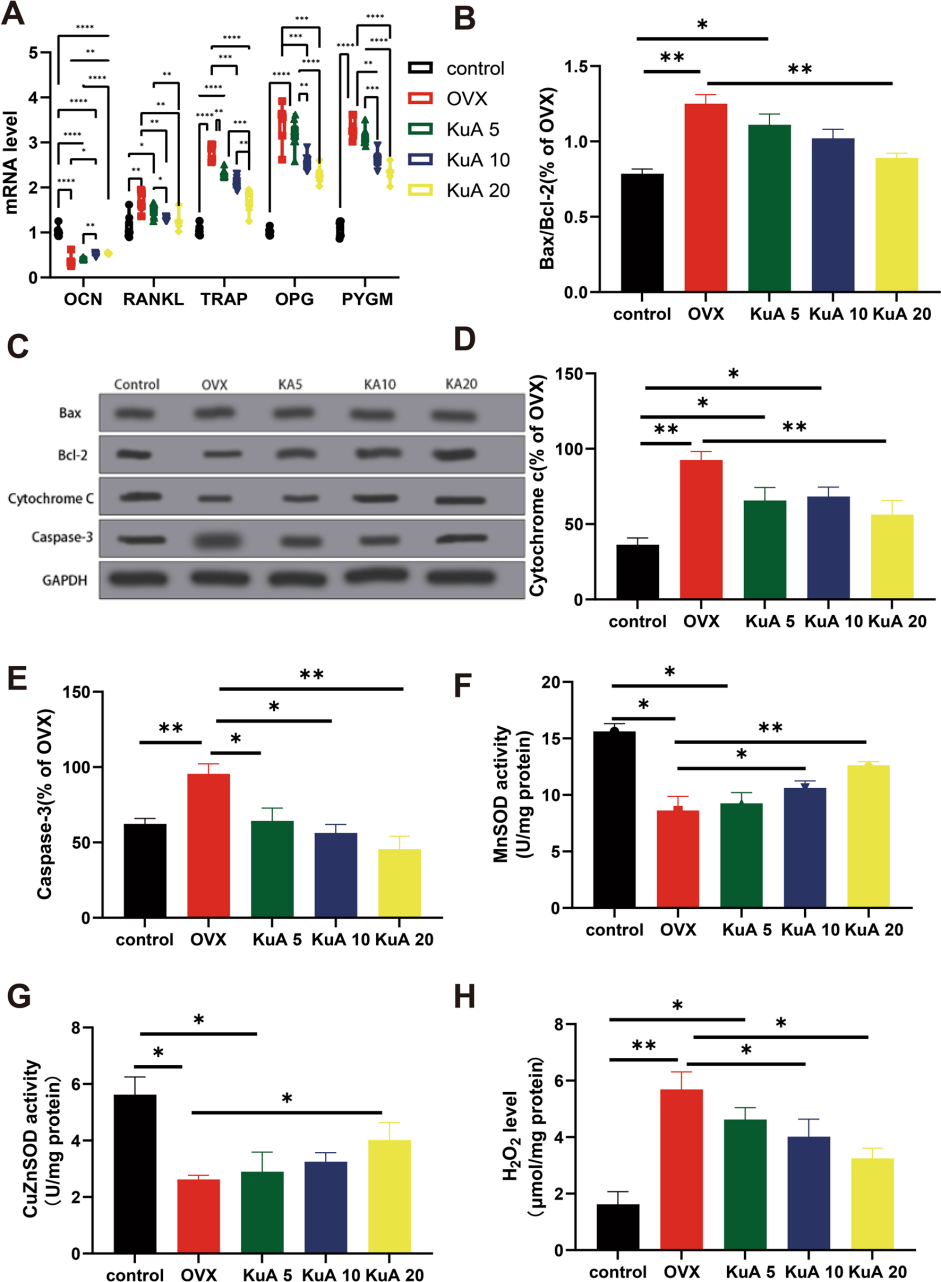
KuA protects against cell apoptosis and oxidative stress level in OVX mice. **A** mRNA expression in the tibia. **B** Bax/BCL-2 level. **C** The original membrane of the western blotting. **D** cytochrome c level. **E** Caspase-3. **F** MnSOD. **G** CuZnSOD. **H** H_2_O_2_ level. *P < 0.05, **P < 0.01,***P < 0.001,****P < 0.0001. KuA: Kukoamine A; OVX: ovariotomy; *Bax*: BCL2 Associated X, Apoptosis Regulator; *BCL2*: BCL2 Apoptosis Regulator; SOD, Superoxide Dismutase; MDA: malondialdehyde

Figure 8A showed the mRNA expression in tibia treated with different concentrations of KuA in OVX mice. We first analyzed the expression of osteogenesis-related genes and found that KuA significantly up-regulated *OCN* expression compared with the OVX group. What’s more, KuA treatment can down-regulate the expression of osteoclast-related genes such as *RNAKL, TRAP, and OPG* than the OVX group. Finally, the *PYGM* was also effectively inhibited in KuA group than the OVX group.

### KuA protects against OVX-induced inflammation

KuA can also prevent inflammation in hippocampal Neurogenesis (Zhang et al. 2017). So we investigated the anti-inflammatory role of KuA administration in the OVX mice and found that *IL-6, CRP, TNF-α*, and *IL-1b* levels were increased significantly in the OVX group than in the control group. Additionally, KuA treatment can inhibit inflammation levels such as *IL-6, CRP, TNF-α*, and *IL-1b* than the OVX group (Fig. 8A).

### KuA protects against OVX-induced cell apoptosis and oxidative stress level

**Fig. 9:**
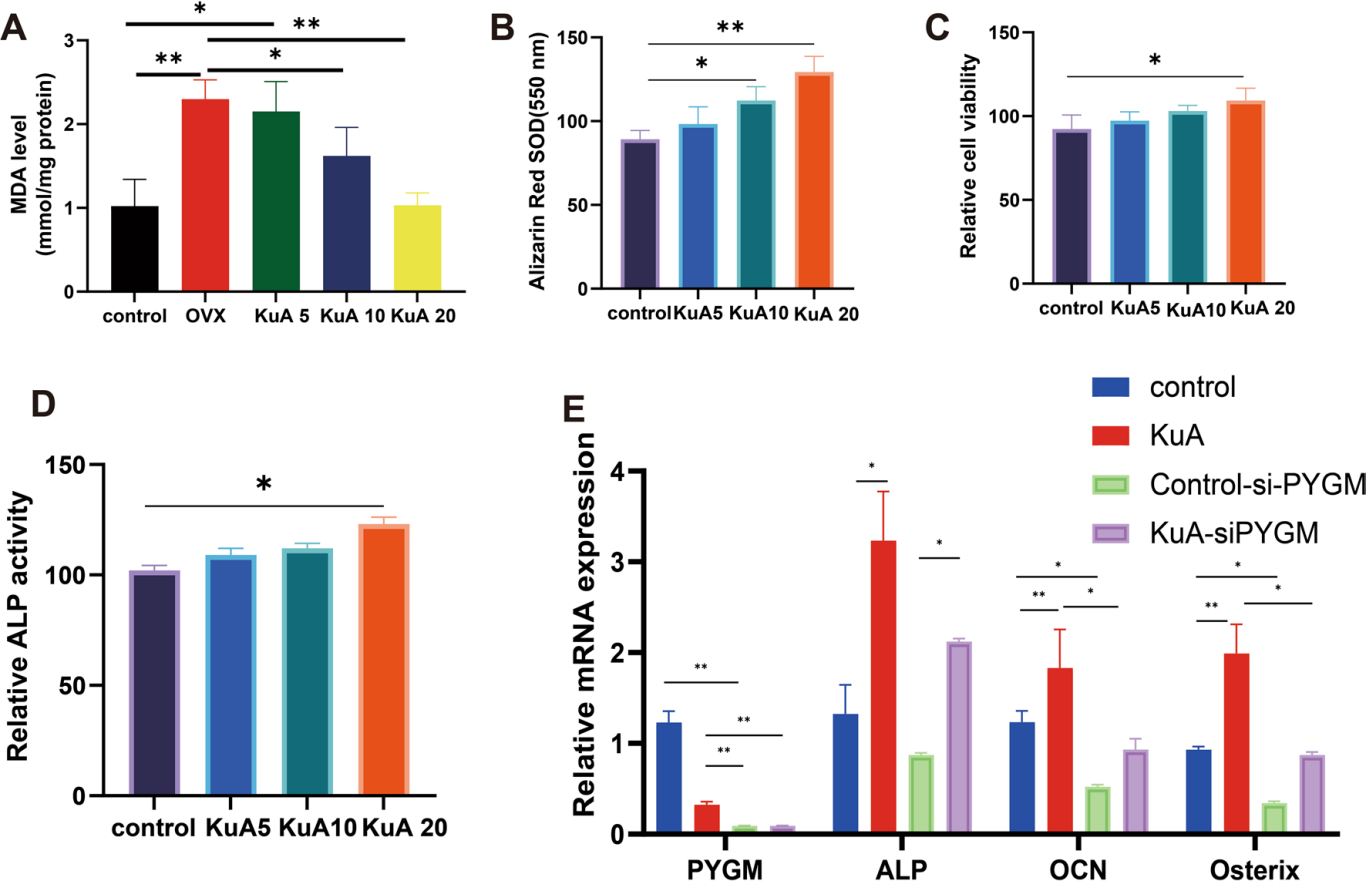
KuA increased the osteoblastic differentiation and mineralized nodule formation of osteoblastic MC3T3-E1 cells. **A** MDA. **B** Relative ALP activity. **C** Alizarin Red SOD. **D** Relative cell viability. **E** mRNA level in MC3T3-E1 cells. *P < 0.05, **P < 0.01,***P < 0.001,****P < 0.0001. KuA: Kukoamine A; OVX: ovariotomy; *ALP*, Alkaline Phosphatase; *PYGM*, Glycogen Phosphorylase, Muscle Associated; *OCN*, osteocalcin; *Osterix*: Sp7 Transcription Factor

According to the previous study, the neuroprotective effects of KuA inhibited oxidative stress in brain injury (Zhang et al. 2016). In this study, we analyzed the effect of KuA administration on cellular processes such as apoptosis and oxidative stress in the OVX mice. The Bax/Bcl-2, cytochrome c, and caspase-3 levels were lower significantly in the control group and KuA administration with 20 mg group than in the OVX group. In addition, we also analyzed the MnSOD activity, CuZnSOD, H_2_O_2,_ and MDA levels, and it showed that the OVX group can increase MnSOD activity and CuZnSOD levels but reduce H_2_O_2_ and MDA levels than the control group. After treatment with KuA, MnSOD activity and CuZnSOD levels increased significantly than OVX group (Figs. 8B–H, 9A).

### KuA increased osteoblastic differentiation and formation of mineralized nodules in MC3T3-E1 cells

We further examined the effects of KuA on osteoblast differentiation such as cell proliferation, matrix maturation, and matrix mineralization in the MC3T3-E1 cell line (Rutkovskiy et al. 2016). Several conventional methods have been used to assess the effects of KuA on osteoblast differentiation, including ALP activity, cell viability, and mineralization. ALP activity plays a key role in new bone mineralization and osteoblast differentiation (Watts 1999; Liu et al. 2014). ALP activity in KuA treatment cells (20 μm) was increased significantly at five days but administration with 5 and 10 μm KuA had no significant difference at five days. After 5 days of incubation with KuA (5, 10, and 20 μM), cell viability improved significantly compared with the control group.

The bone matrix is mineralized by osteoblast differentiation, leading to the induction of calcium and phosphorus-based minerals. Therefore, bone mineralization develops with different matrix proteins. (Owen and Reilly 2018). Alizarin Red S is a commonly used histochemical method to assess calcium-rich deposits in osteoblast mineralization (Virtanen and Isotupa 1980). After simultaneous treatment with KuA (20 μM) and induction reagent, positive colonies stained with Alizarin Red S were larger than untreated control cells. These results suggested that KuA promotes osteoblast differentiation and the formation of mineralized nodules (Fig. 9).

### Transient transfections of siRNA molecules

The increased differentiation of osteoblasts is closely related to the high expression of the main osteoblast marker gene *ALP, OCN,* and *Osterix*. Osteoblastic MC3T3-E1 cell line treated with 20 μM of KuA significantly increased the expression of *OCN* and *Osterix* compared to the OVX group. In addition, the *PYGM* mRNA level was significantly lower in the OVX group than in the control group and after treatment with KuA, the *PYGM* mRNA level decreased significantly than OVX group (Fig. 9E).

To further explore the *PYGM* gene functions in osteoblast differentiation, transient transfections of *PYGM* siRNA molecules were investigated and we found that KuA reduced the *PYGM* mRNA expression level. After transfection of *PYGM* siRNA, the *OCN* and *Osterix* mRNA levels decreased significantly than the control group. In addition, after treatment with KuA, we found that the KuA with *PYGM* siRNA group decreased the *OCN*, and *Osterix* mRNA levels significantly than the *PYGM* siRNA group (Fig. 9E).

Finally, to further clarify the influence of KuA intervention on bone loss, we further verified that KuA intervention would significantly improve the progress by principal cause analysis.

## Discussion

OP is a systemic bone disease characterized by decreased bone mass and deterioration of bone microarchitecture with associated bone fragility and increased risk of fracture (Ensrud and Crandall 2017). Clinical vertebral and femoral fractures are the most devastating consequences of OP and are associated with morbidity and mortality (Black and Rosen 2016; Rachner et al. 2011). The mechanism of OP is related to multiple factors such as the regulation by the adaptive immune response (Weitzmann and Ofotokun 2016), genetic determination (Yang et al. 2020), oxidative stress, apoptotic mechanisms, sex-steroid deficiency, and macroautophagy (Hendrickx et al. 2015). In our study, the firstly obvious finding to emerge from the analysis was that 22 active ingredients of LC we investigated in treatment with OP were associated with a variety of proteins and signaling pathways, indicating that active ingredients play a potential role in the development of OP. And then we found that KuA plays important role in the treatment of OP through *PYGM* pathway. In vivo and in vitro experiments, we also found that KuA improves bone loss via inflammation and oxidative stress.

The role of LC has been widely studied and has been confirmed to exert beneficial effects on improving insulin resistance, lipid metabolism, bone metabolism, and tumor progress by inhibiting inflammation and immunity (Jeong et al. 2012; Park et al. 2014; Ye et al. 2008). The bioactive ingredients included KuA and B, scopolin, aurantiamide acetate, and others which have been uploaded into Table 1. The IC50 values of kukoamines A and B were 11.4, 9.5, respectively (Jiang et al. 2020). There are also significant differences in the extraction processes of the two drugs. What’s more, kukoamine A and B, were comparatively investigated for their antioxidant and cytoprotective effects in Fenton-damaged bone marrow-derived mesenchymal stem cells (bmMSCs). When compared with kukoamine B, kukoamine A consistently demonstrated higher IC50 values in PTIO-scavenging (pH 7.4), Cu^2+^ -reducing, DPPH-scavenging, O_2_^−^ -scavenging, and OH− scavenging assays. However, in the PTIO-scavenging assay, the IC50 values of each kukoamine varied with pH value. In the Fe2 + -chelating assay, kukoamine B presented greater UV–Vis absorption and darker color than kukoamine A. In the HPLC^−^ESI^−^MS/MS analysis, kukoamine A with DPPH produced radical-adduct-formation (RAF) peaks (m/z 922 and 713). The 3- (4,5-Dimethylthiazol-2-yl)-2,5-diphenyl (MTT) assay suggested that both kukoamines concentration-dependently increased the viabilities of Fenton-damaged bmMSCs at 56.5–188.4 μM (Li et al. 2018). In particular, it protects the liver from lipid degeneration (Chen et al. 2018). Administration of aurantiamide acetate suppresses the growth gliomas by blocking autophagic flex (Yang et al. 2015). Scopolin also contains bioactive components used to treat and prevent OP (Park et al. 2020). Anxiolytic and anticonvulsant potential of stigmasterol have the positive modulation of GABA receptors and were considered to be candidates for steroidal drugs in the treatment of neurological disorders (Karim et al. 2021). A total of 22 therapeutic compounds have varying degrees of therapeutic effects on OP, mediated by a variety of cytokines and signaling pathways.

For OP, LC extract prevents OVX-induced BMD loss in mice by promoting osteoblast differentiation (Park et al. 2014). In addition, Kukoamine B has anti-osteoporotic effects in osteoblasts, osteoclasts, and ovariectomized OP mouse models (Park et al. 2019b). What’s more, a combined extract of Lycii Radicis and Achyranthes japonica also has an anti-osteoporotic effect (Park et al. 2019a). However, there are few studies to analyze which active components in LC may play an important role in OP. A previous study proved the role of LC-derived substances KuA and KuB in inhibiting amyloid aggregation in Alzheimer’s disease and type II diabetes (Jiang et al. 2020). These findings suggested that several LC active ingredients have synergistic effects in the treatment of OP. The active ingredients in LC such apigenin and scopolin were indeed found to possess anti-osteoporotic effects in the previous studies (Park et al. 2020; Tantowi et al. 2020), but due to there are many active components in LC, further studies are needed to identify the main anti-osteoporotic active ingredients at present and based on this, further analysis of the anti-osteoporosis effect of KuA by Vivo experiment via dual-energy X-ray absorptiometry, Micro-CT analysis, biomechanical analysis, Western blotting, and PCR analysis. Therefore, these integrated, complex methods were better to understand the role of KuA anti-osteoporotic bioactive ingredients in LC. However, which bioactive ingredients were the best ingredients in anti-osteoporotic progress also needs to be further in the future study.

Besides, there are also obvious differences in the anti-osteoporosis effects of different LC components such as Kukoamine A, Kukoamine B, apigenin, and scopolin. Kukoamine B was found to have anti-osteoporotic effects in promoting osteoblast differentiation but did not affect osteoclast differentiation, and ovariectomized OP mouse models in the previous studies (Park et al. 2019b). apigenin is comparable to diclofenac in suppressing inflammation and catabolic proteases for osteoporotic-osteoarthritis prevention (Tantowi et al. 2020). Scopolin treatment enhanced alkaline phosphatase activity and increased mineralized nodule formation in MC3T3-E1 pre-osteoblastic cells. However, osteoclast differentiation in primary-cultured monocytes was reduced by treatment with scopolin. Consistently, scopolin treatment increased osteoblast differentiation in the co-culture of monocytes (osteoclasts) and MC3T3-E1 (osteoblast) cells. Scopolin treatment prevented bone mineral density loss in OVX-induced osteoporotic mice. These results suggest that scopolin could be a therapeutic bioactive constituent for the treatment and prevention of osteoporosis (Park et al. 2020). However, our study also found that Kukoamine A had significant effects on osteoblasts and osteoclasts. Therefore, different LC components have different anti-osteoporosis effects.

Analysis of GO and KEGG enrichment revealed that steroid hormone, oxygen levels, and lipid metabolism may be the mechanism of LC in treating OP, which is according to previous research (Gennari et al. 2007; Domazetovic et al. 2017). The pathways with the best correlation were selected here for a discussion on the mechanism of the LC treatment for OP. Our results indicated a potential mechanism for the treatment of LC with OP.

As can be seen from the network of potential LC-OP target genes, many target genes can be regulated by β bonds, including but not limited to *PYGM, RBM47, VNN1, TSFM*, and *ICAM1*. An online meta-analysis examining polygene expression profiles in women finds that *PYGM* is associated with BMD (He et al. 2016). These results indicated that LC has the biological characteristics of multi-component and multi-target in the treatment of OP. In addition, the PPI results showed that the 89 target proteins were not independent of each other, but were linked and interacted with each other (Zhang et al. 2019b). These results also suggested that LC may participate in the remission and treatment of OP by regulating various proteins, and KuA may be the most critical target.

CB-Dock was designed to perform blind docking at predicted sites, instead of the entire surface of a protein. Therefore, the first step is to detect putative binding sites (Cavity detection). Since the ligand binding sites are usually larger cavities, we select several top cavities according to cavity size for further analysis (Cavity sorting). Then, we calculate the docking center and adjust the docking box size. These parameters are required for molecular docking with AutoDock Vina (Center and Size). After the docking process is finished, the bound poses are reranked according to the docking score (Dock and Rerank). The first conformation is considered the best binding pose and the corresponding site is the optimal binding site for the query ligand (Liu et al. 2020; Cao and Li 2014).

As previously mentioned, KuA is a major bioactive component extracted from the root barks of LC which can upregulate Srebp-1c and inhibit insulin-stimulated glucose uptake and lipid accumulation in hepatic steatosis (Li et al. 2017). It also has an anti-oxidative effect and anti-apoptosis stress in protecting the brain against injury by pMACO oxidative effect and anti-apoptosis stress (Liu et al. 2017). We further investigated the effects of KuA on osteoporotic mice and cell lines. Radiographic results and mechanical tests showed that KuA significantly improved the bone microstructure and mechanical strength of osteoporotic mice and osteoblast activity.

Inflammation status regulated the progress of OP in many previous studies (Cortet et al. 2019; Lee and Kim 2020; Zhou et al. 2021b). In the absence of estrogen, fracture healing is hindered by an increase in proinflammatory cytokines such as *IL-6*, which may lead to poor healing (Fischer and Haffner-Luntzer 2021). The progressive increase in the secretion of *IL-1β* and *TNF-α* contributes to postmenopausal bone loss (Pacifici et al. 1993; Chow et al. 2020). In previous studies, KuA attenuated the pro-inflammatory cytokines such as *IL-1β* and *TNF-a* levels in radiation-induced neuroinflammation (Zhang et al. 2017). There are similarities between the attitudes expressed in our study and found that KuA alleviated the inflammation level in treatment OP.

Oxidative stress is thought to be a causative factor in many disease states, possibly including a reduction in bone mineral density in OP (Kimball et al. 2021). Oxidative stress serves as potential biomarkers such as superoxide dismutase (SOD) in erythrocytes, catalase (CAT), total antioxidant status (TAS), hydroperoxides (HY), advanced oxidation protein products (AOPP), malondialdehyde (MDA), and vitamin B12 (VB12) in the etiopathophysiology and clinical course of OP (Zhou et al. 2016). Following the present results, previous studies have demonstrated that KuA has the ability to anti-oxidative stress via attenuated LDH release, ROS production, MDA level, MMP loss, and intracellular Ca2 + overload (Hu et al. 2015). These results are consistent with our studies that KuA reduces the oxidative stress level in OP.

Cell apoptosis was also involved in the progress of OP (Gruver-Yates and Cidlowski 2013). Expression of the pro-apoptotic factor caspase-3 or the anti-apoptotic factor *Bcl-2* has been shown to affect osteoblast apoptosis (Akiyama et al. 2003). OP reduces the expression of caspase-3 and *Bcl-2* in osteoblasts, thereby preventing bone loss (Jilka et al. 2014). Mitochondrial dysfunction in osteoblasts contributes to glucocorticoid-induced bone loss (Chen et al. 2020). *Bcl-2*-associated X protein (*Bax*) is a critical executioner of mitochondrial regulated cell death through its lethal activity of permeabilizing the mitochondrial outer membrane (Spitz and Gavathiotis 2022). The *BCL2* family proteins comprise the sentinel network that regulates the mitochondrial or intrinsic apoptotic response (Hata et al. 2015). KuA inhibited apoptosis induction by decreasing the level of Bax, and caspase-3 in human glioblastoma cell growth (Wang et al. 2016). Their results match the observation in our study that KuA attenuated the cell apoptosis level in OP.

Glycogen Phosphorylase (*PYGM*) is a key enzyme in the first step of glycogenolysis, encoding the muscle-specific glycogen phosphorylase (myophosphorylase). The main role of *PYGM* is to provide sufficient energy for muscle contraction. However, it is expressed in tissues other than muscle, such as the bone, brain, lymphoid tissues, and blood. *PYGM* also played an important role in a variety of diseases such as early fatigue, myalgia, and contractures (Villarreal-Salazar et al. 2021; Gomes et al. 2020; Jin and Yang 2019; Nogales-Gadea et al. 2015). *PYGM* was identified as a candidate gene that may play an important role in BMD regulation in women (He et al. 2016). *PYGM* plays a potential role in glycogenolysis which affects glycogen metabolism, skeletal muscle, and bone metabolism (He et al. 2016; Tarnopolsky 2018). In our study, *PYGM* also play important role in KuA treatment of OP. In vivo and in vitro experiments, the *PYGM* mRNA level was regulated by KuA. What’s more, to further clarify the role of *PYGM* in KuA in the treatment of OP, we used transient transfections of siRNA molecules to study the role of *PYGM* in the treatment of OP. Interestingly, *PYGM* may be a novelty discovered biologically meaningful functional modules in the progress of KuA treatment of OP.

There are some limitations in our study. Kukoamine B was found to have anti-osteoporotic effects in promoting osteoblast differentiation but did not affect osteoclast differentiation, and ovariectomized OP mouse models in previous studies (Park et al. 2019a). These results suggest that Kukoamine B may be a potential therapeutic candidate for the treatment of osteoporosis. However, in our study, Kukoamine A was found to be superior to Kukoamine B in terms of oral bioavailability (OB), drug-likeness (DL), intestinal epithelial permeability, blood–brain barrier penetrability, and water solubility, which needs to be further validated using experiments. Besides, In this study, we chose autophagy-related proteins to analyze changes in autophagy levels, but additional autophagy-related assays, including flow cytometry, are needed in future studies.

## Conclusion

Taken together, KuA extract from LC has obvious advantages in the treatment of OP. The biological activity of the active substance of LC and the signal transduction pathway of the target OP gene were studied by network pharmacology method and molecular binding test. Meanwhile, this is the first study to investigate the anti-osteoporotic effect of KuA in vivo and vitro. The results suggest that KuA could be a good candidate for treating and preventing OP.

## Supplementary Information

**Additional file 1:** **Figure S1.** The workflow of KuA in treatment of OP. **Figure S2.** GO enrichment analysis among target genes. (A) Cell type signature. (B) Disgenet analysis. (C) Trrust analysis. (D) Pagenbase analysis. (E) Transcription factor. **Figure S3.** KuA improve the mechanical properties and inflammation level in OVX mice. (A) Stiffness of tibia. (B) Displacement of tibia. (C) Energy absorption. (D) serum IL-6. (E)serum CRP. (F) serum TNF-α. (G) serum IL-1b. (H) Principal genetic analysis among all variable. **Figure S4.** The PYGM related signaling pathway of potential target genes of LC in OP. **Figure S5.** KuA increased significantly the tibia and spine bone microstructure and mechanical properties in OVX mice. **Figure S6.** KuA increased significantly the spine bone microstructure and mechanical properties in OVX mice. **Table S1.** Primer Sequences used for RT-QPCR.

## Data Availability

The data used to support the findings of this study are included within the article.
