# Correction: Identification of kukoamine a as an anti-osteoporosis drug target using network pharmacology and experiment verification

**DOI:** 10.1186/s10020-023-00654-1

**Published:** 2023-04-21

**Authors:** Liying Luo, Zhiyuan Guan, Xiao Jin, Zhiqiang Guan, Yanyun Jiang

**Affiliations:** 1 Department of Ophthalmology, Tongren Hospital, Shanghai Jiao Tong University School of Medicine, Shanghai, China; 2 Department of Orthopedics, The Shanghai Tenth People’s Hospital of Tongji University, Shanghai, China; 3 Department of Rheumatology and Immunology, The First People’s Hospital of Xuzhou, Xuzhou, 221002 Jiangsu People’s Republic of China; 4 Department of Dermatology, Xuzhou Municipal Hospital Affiliated With Xuzhou Medical University, Xuzhou, 221002 Jiangsu People’s Republic of China

Correction: Molecular Medicine (2023) 29:36 10.1186/s10020-023-00625-6

Following publication of the original article (Luo et al. 2023), we have been informed that the corresponding authors were incorrectly marked.

The correspondence has been updated above original article has been corrected.
