# Supplementary material for: Identification of kukoamine a as an anti-osteoporosis drug target using network pharmacology and experiment verification
Source: Mol Med. 2023 Mar 20;29:36. doi: 10.1186/s10020-023-00625-6 (PMC10029210; doi:10.1186/s10020-023-00625-6)
Supplement: Supplementary file 1 — Additional file 1: Figure S1. The workflow of KuA in treatment of OP. Figure S2. GO enrichment analysis among target genes. (A) Cell type signature. (B) Disgenet analysis. (C) Trrust analysis. (D) Pagenbase analysis. (E) Transcription factor. Figure S3. KuA improve the mechanical properties and inflammation level in OVX mice. (A) Stiffness of tibia. (B) Displacement of tibia. (C) Energy absorption. (D) serum IL-6. (E)serum CRP. (F) serum TNF-α. (G) serum IL-1b. (H) Principal genetic analysis among all variable. Figure S4. The PYGM related signaling pathway of potential target genes of LC in OP. Figure S5. KuA increased significantly the tibia and spine bone microstructure and mechanical properties in OVX mice. Figure S6. KuA increased significantly the spine bone microstructure and mechanical properties in OVX mice. Table S1. Primer Sequences used for RT-QPCR. [file 10020_2023_625_MOESM1_ESM.docx]

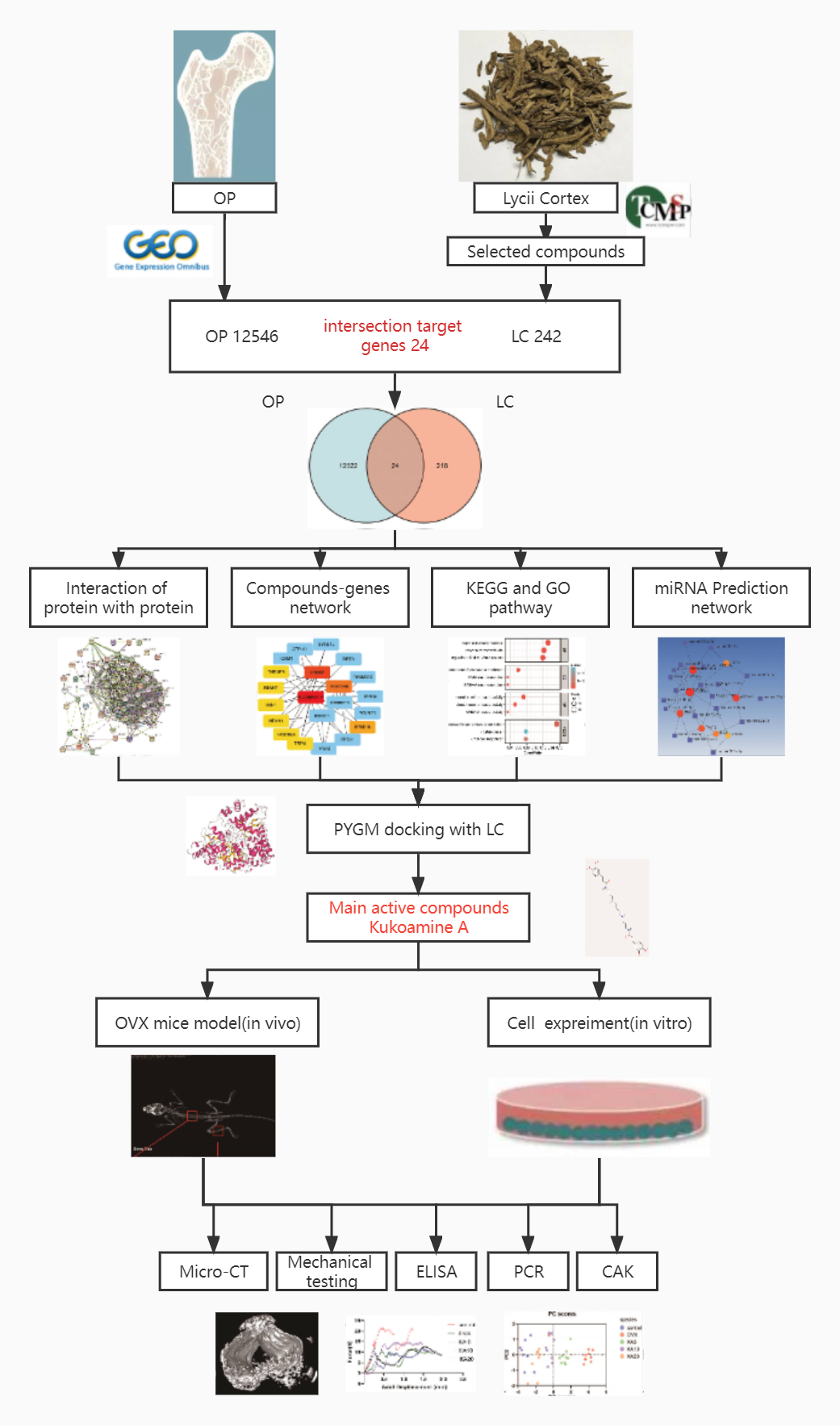


Figure S1. The workflow of KuA in treatment of OP.


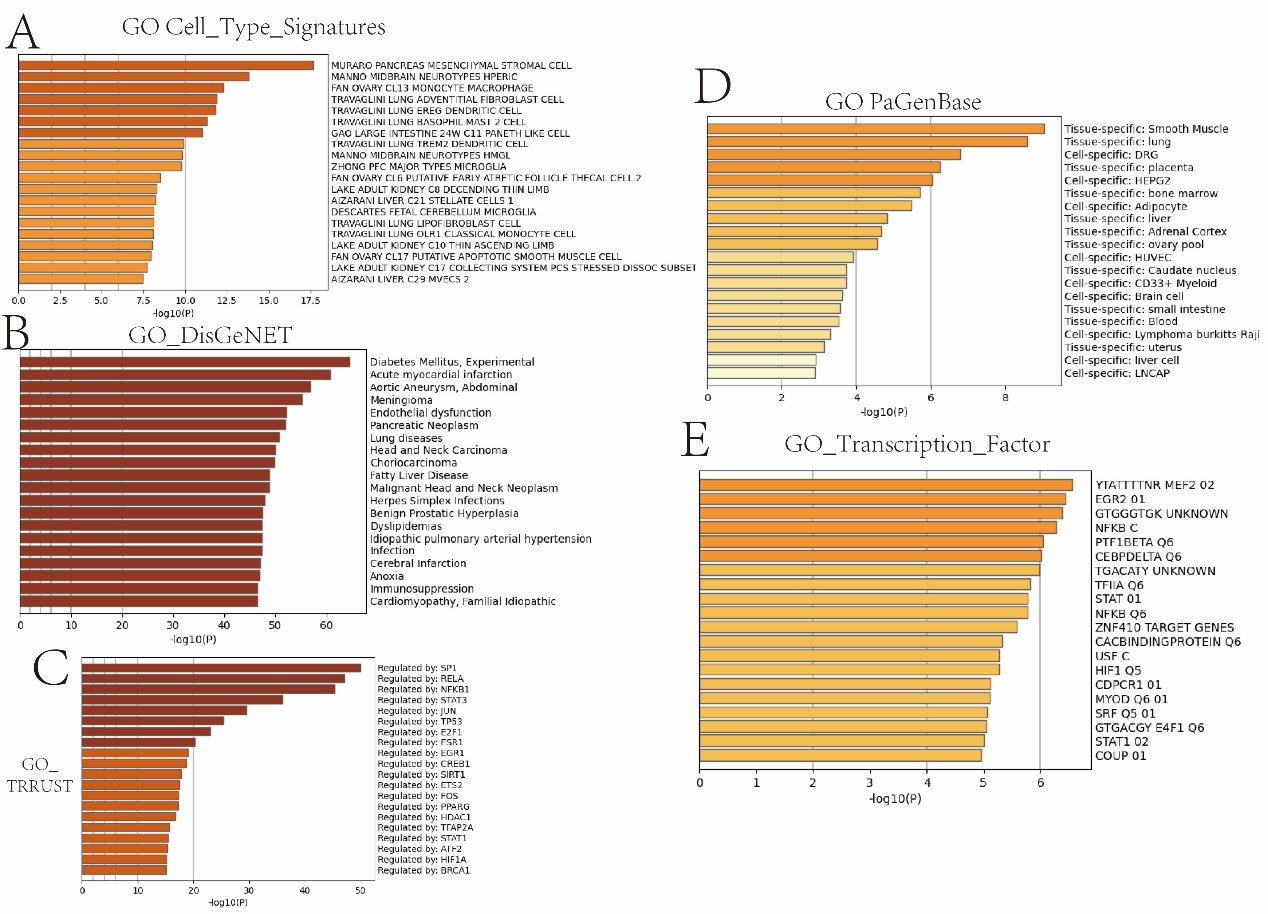


Figure S2. GO enrichment analysis among target genes. (A) Cell type signature. (B) Disgenet analysis. (C) Trrust analysis. (D) Pagenbase analysis. (E) Transcription factor.


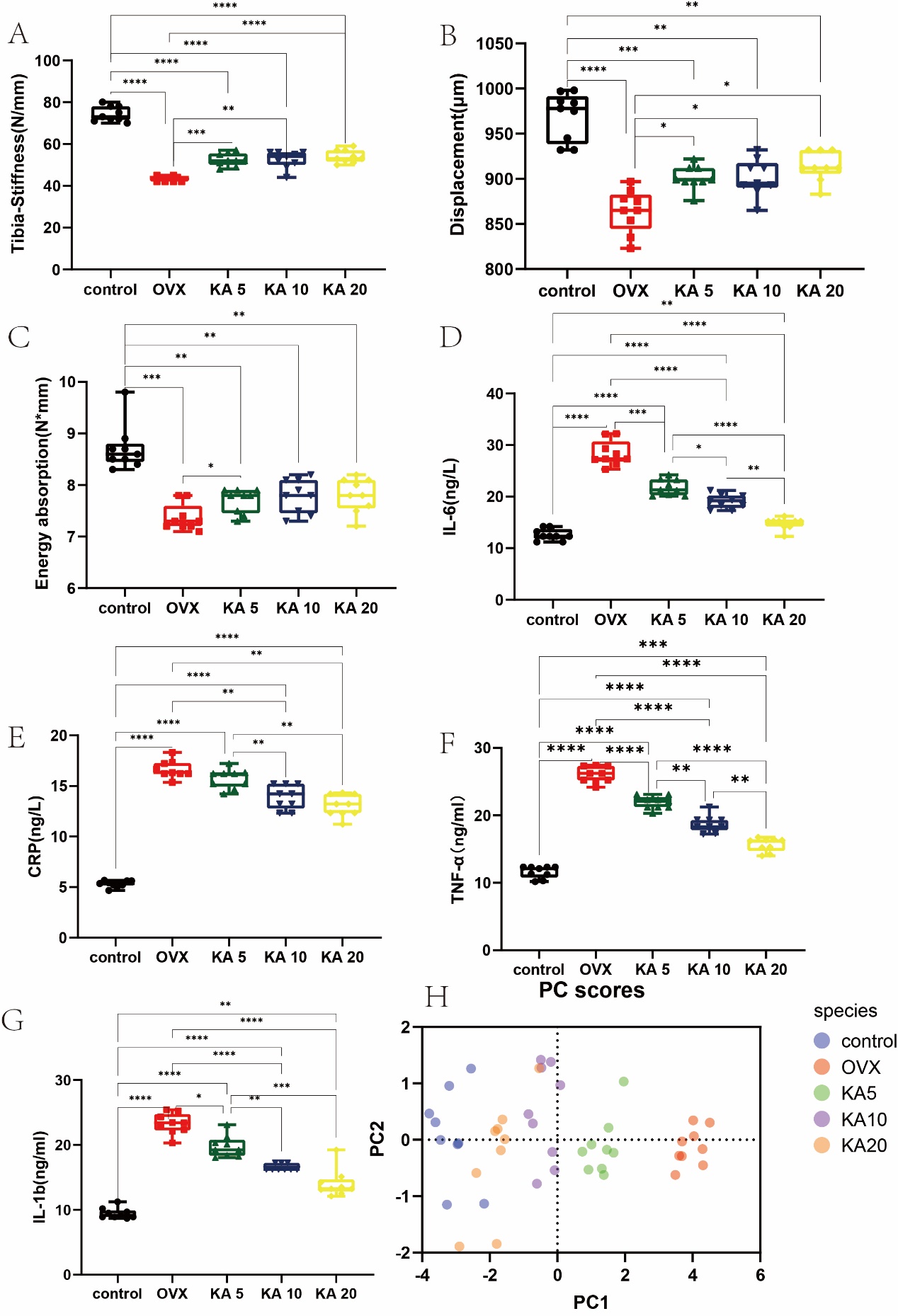


Figure S3. KuA improve the mechanical properties and inflammation level in OVX mice. (A) Stiffness of tibia. (B) Displacement of tibia. (C) Energy absorption. (D) serum IL-6.(E)serum CRP. (F) serum TNF-α. (G) serum IL-1b.(H) Principal genetic analysis among all variable.


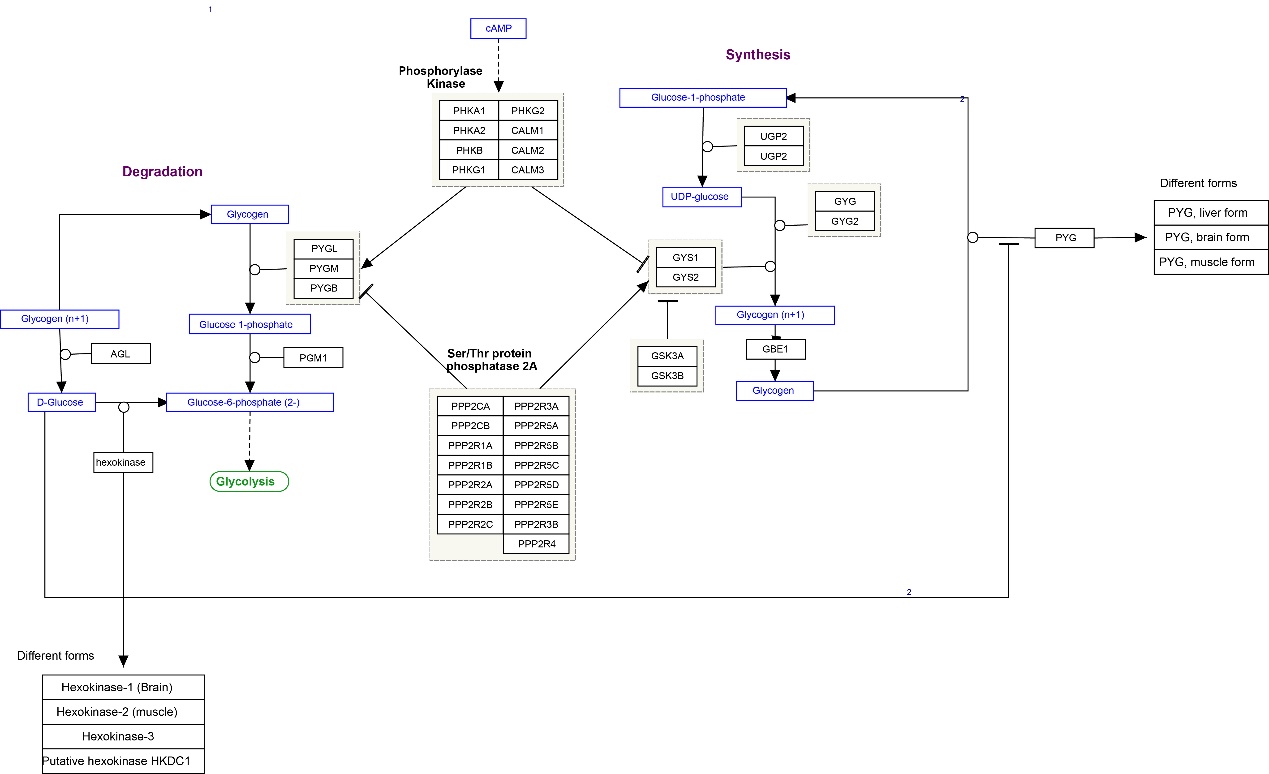


Figure S4. The PYGM related signaling pathway of potential target genes of LC in OP.


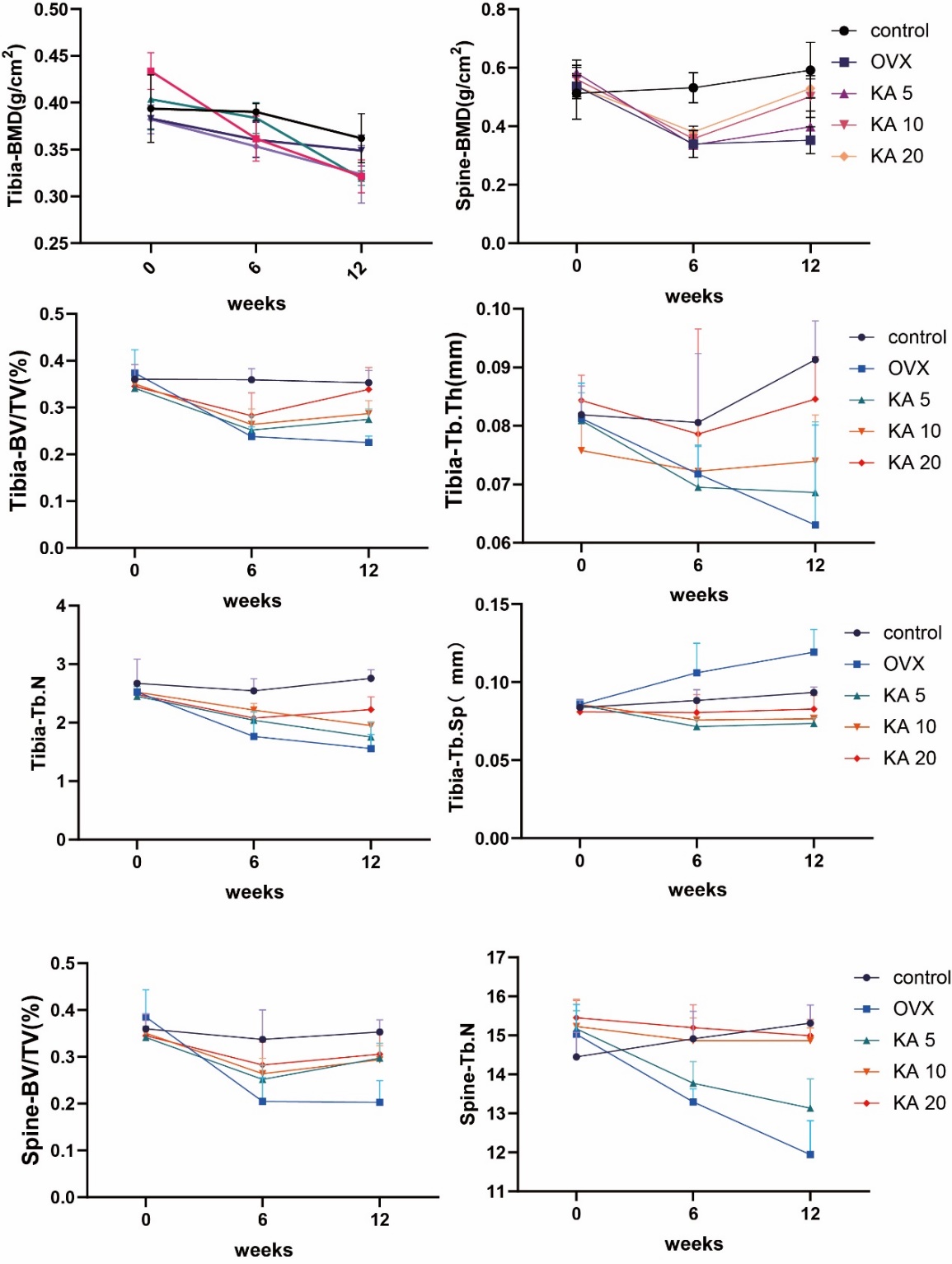


Figure S5. KuA increased significantly the tibia and spine bone microstructure and mechanical properties in OVX mice.


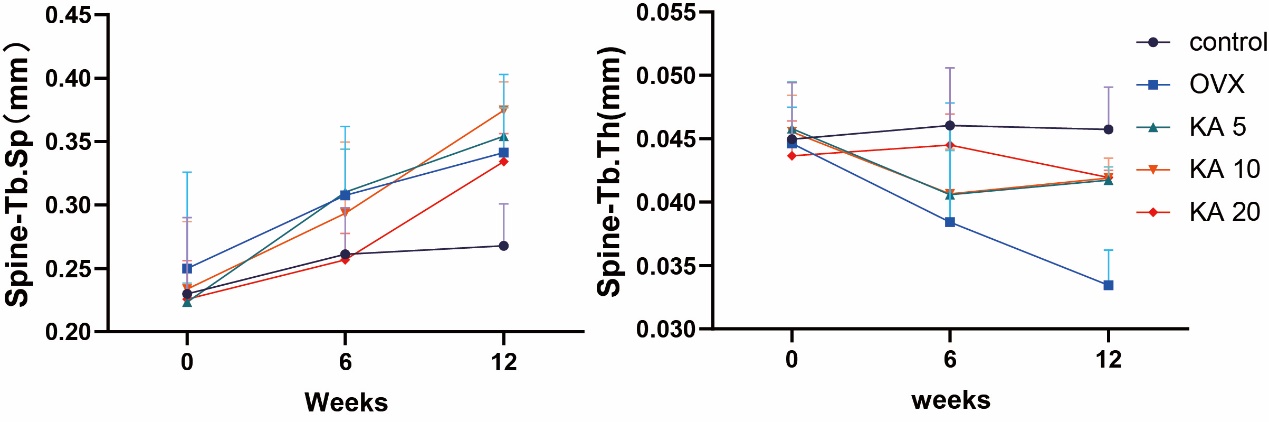


Figure S6. KuA increased significantly the spine bone microstructure and mechanical properties in OVX mice.

| Table S1: Primer Sequences used for RT-QPCR | | |
| --- | --- | --- |
|  | Forward primer sequences | Reverse primer sequences |
| OCN | 5'-AAGCAGGAGGGCAATAAGGT-3' | 5'- TAG GCGGTCTTCAAGCCAT-3' |
| RANKL | 5'-GCGCAACAGTGTTTCCACAG-3' | 5'-CACGCTTGGATCACAGTAAGG-3' |
| OPG | 5'-GAGGAGTCTGGTAGTGGTTCC-3' | 5'-GGGCGTTTCGTTGAATATGCG-3' |
| osterix | 5'-CCACCAAGAATTACTCAGAACC-3' | 5'-AAGGACCGACTCACTCAGTCT-3' |
| Alp | 5'-TGGTTACTGCTGATCATTCCCACG-3' | 5'-AATGTAGTTCTGCTCATGGACGCC-3' |
| IL-6 | 5'-GGCGGATCGGATGTTGTGAT-3' | 5'-GGACCCCAGACAATCGGTTG-3' |
